# Non‐vitamin‐K‐antagonist oral anticoagulants (NOACs) after acute myocardial infarction: a network meta‐analysis

**DOI:** 10.1002/14651858.CD014678.pub2

**Published:** 2024-01-24

**Authors:** Samer Al Said, Klaus Kaier, Wael Sumaya, Dima Alsaid, Daniel Duerschmied, Robert F Storey, C. Michael Gibson, Dirk Westermann, Samer Alabed

**Affiliations:** Department of Cardiology and Angiology, University Heart Center Freiburg Bad KrozingenFaculty of Medicine, University of FreiburgFreiburgGermany; Institute for Medical Biometry and StatisticsFaculty of Medicine and Medical Center, University of FreiburgFreiburgGermany; Department of Medicine, Faculty of MedicineDalhousie University, QE II Health Sciences Centre, Halifax InfirmaryHalifaxCanada; Institute for Evidence in Medicine, Medical Center – University of FreiburgFaculty of Medicine, University of FreiburgFreiburgGermany; Department of Cardiology, Angiology, Haemostaseology and Medical Intensive CareUniversity Medical Centre Mannheim, Medical Faculty Mannheim, Heidelberg UniversityMannheimGermany; European Center for AngioScience (ECAS) and German Center for Cardiovascular Research (DZHK) partner site Heidelberg/Mannheim, Mannheim, GermanyMannheimGermany; Department of Infection, Immunity and Cardiovascular DiseaseUniversity of SheffieldSheffieldUK; Cardiology Division, Beth Israel Deaconess Medical CenterHarvard Medical SchoolBostonMAUSA

**Keywords:** Humans, Anticoagulants, Dabigatran, Hemorrhage, Myocardial Infarction, Network Meta-Analysis, Platelet Aggregation Inhibitors, Rivaroxaban

## Abstract

**Background:**

Balancing the risk of bleeding and thrombosis after acute myocardial infarction (AMI) is challenging, and the optimal antithrombotic therapy remains uncertain. The potential of non‐vitamin K antagonist oral anticoagulants (NOACs) to prevent ischaemic cardiovascular events is promising, but the evidence remains limited.

**Objectives:**

To evaluate the efficacy and safety of non‐vitamin‐K‐antagonist oral anticoagulants (NOACs) in addition to background antiplatelet therapy, compared with placebo, antiplatelet therapy, or both, after acute myocardial infarction (AMI) in people without an indication for anticoagulation (i.e. atrial fibrillation or venous thromboembolism).

**Search methods:**

We searched CENTRAL, MEDLINE, Embase, the Conference Proceedings Citation Index – Science, and two clinical trial registers in September 2022 with no language restrictions. We checked the reference lists of included studies for any additional trials.

**Selection criteria:**

We searched for randomised controlled trials (RCTs) that evaluated NOACs plus antiplatelet therapy versus placebo, antiplatelet therapy, or both, in people without an indication for anticoagulation after an AMI.

**Data collection and analysis:**

Two review authors independently checked the results of searches to identify relevant studies, assessed each included study, and extracted study data. We conducted random‐effects pairwise analyses using Review Manager Web, and network meta‐analysis using the R package 'netmeta'. We ranked competing treatments by P scores, which are derived from the P values of all pairwise comparisons and allow ranking of treatments on a continuous 0‐to‐1 scale.

**Main results:**

We identified seven eligible RCTs, including an ongoing trial that we could not include in the analysis. Of the six RCTs involving 33,039 participants, three RCTs compared rivaroxaban with placebo, two RCTs compared apixaban with placebo, and one RCT compared dabigatran with placebo. All participants in the six RCTs received concomitant antiplatelet therapy.

The available evidence suggests that rivaroxaban compared with placebo reduces the rate of all‐cause mortality (risk ratio (RR) 0.82, 95% confidence interval (CI) 0.69 to 0.98; number needed to treat for an additional beneficial outcome (NNTB) 250; 3 studies, 21,870 participants; high certainty) and probably reduces cardiovascular mortality (RR 0.83, 95% CI 0.69 to 1.01; NNTB 250; 3 studies, 21,870 participants; moderate certainty). There is probably little or no difference between apixaban and placebo in all‐cause mortality (RR 1.09, 95% CI 0.88 to 1.35; number needed to treat for an additional harmful outcome (NNTH) 334; 2 studies, 8638 participants; moderate certainty) and cardiovascular mortality (RR 0.99, 95% CI 0.77 to 1.27; number needed to treat not applicable; 2 studies, 8638 participants; moderate certainty). Dabigatran may reduce the rate of all‐cause mortality compared with placebo (RR 0.57, 95% CI 0.31 to 1.06; NNTB 63; 1 study, 1861 participants; low certainty). Dabigatran compared with placebo may have little or no effect on cardiovascular mortality, although the point estimate suggests benefit (RR 0.72, 95% CI 0.34 to 1.52; NNTB 143; 1 study, 1861 participants; low certainty).

Two of the investigated NOACs were associated with an increased risk of major bleeding compared to placebo: apixaban (RR 2.41, 95% CI 1.44 to 4.06; NNTH 143; 2 studies, 8544 participants; high certainty) and rivaroxaban (RR 3.31, 95% CI 1.12 to 9.77; NNTH 125; 3 studies, 21,870 participants; high certainty). There may be little or no difference between dabigatran and placebo in the risk of major bleeding (RR 1.74, 95% CI 0.22 to 14.12; NNTH 500; 1 study, 1861 participants; low certainty).

The results of the network meta‐analysis were inconclusive between the different NOACs at all individual doses for all primary outcomes. However, low‐certainty evidence suggests that apixaban (combined dose) may be less effective than rivaroxaban and dabigatran for preventing all‐cause mortality after AMI in people without an indication for anticoagulation.

**Authors' conclusions:**

Compared with placebo, rivaroxaban reduces all‐cause mortality and probably reduces cardiovascular mortality after AMI in people without an indication for anticoagulation. Dabigatran may reduce the rate of all‐cause mortality and may have little or no effect on cardiovascular mortality. There is probably no meaningful difference in the rate of all‐cause mortality and cardiovascular mortality between apixaban and placebo. Moreover, we found no meaningful benefit in efficacy outcomes for specific therapy doses of any investigated NOACs following AMI in people without an indication for anticoagulation. Evidence from the included studies suggests that rivaroxaban and apixaban increase the risk of major bleeding compared with placebo. There may be little or no difference between dabigatran and placebo in the risk of major bleeding. Network meta‐analysis did not show any superiority of one NOAC over another for our prespecified primary outcomes.

Although the evidence suggests that NOACs reduce mortality, the effect size or impact is small; moreover, NOACs may increase major bleeding. Head‐to‐head trials, comparing NOACs against each other, are required to provide more solid evidence.

## Plain language summary

**What are the benefits and harms of non‐vitamin K antagonist oral anticoagulants (which help to prevent blood clot formation) after a heart attack**

**Key messages**

• Compared with placebo (dummy treatment), rivaroxaban reduces death from any cause (all‐cause death) and probably reduces death from diseases of the heart and blood vessels (cardiovascular death) after a heart attack. Dabigatran may reduce all‐cause death but may have little or no effect on cardiovascular death. Apixaban is probably no more effective than placebo for reducing all‐cause death or cardiovascular death after a heart attack.

• Apixaban and rivaroxaban increase the risk of major bleeding compared to placebo.

• There is a need for studies that compare non‐vitamin K antagonist oral anticoagulants (NOACs) directly against each other.

**What is heart attack?**

Heart attack is a life‐threatening event that happens when the blood supply to the heart muscle is suddenly interrupted, causing tissue damage. Choosing the best treatment for people after a heart attack remains challenging in clinical practice. Despite treatment with antiplatelet medicines (which prevent platelets from sticking together and forming a blood clot), heart attack survivors are at increased risk of death.

**Why did we do this Cochrane review?**

The aim of this review was to investigate whether adding next‐generation blood thinners (NOACs) to antiplatelet medicines is safe and more effective than antiplatelet medicines alone after a heart attack. NOACs help to prevent blood clot formation by slowing blood clotting time or changing the way in which clotting occurs.

**What did we do?**

We searched for studies that tested the benefits and risks of NOACs in combination with background antiplatelet therapy compared with placebo, antiplatelet therapy, or both, after a heart attack.

**How up‐to‐date is this review?**

We included evidence up to September 2022.

**What did we find?**

We included six studies that involved 33,039 people (two studies compared apixaban with placebo, three studies compared rivaroxaban with placebo, and one study compared dabigatran with placebo). All participants in all studies received antiplatelet medicines. We compared all the NOACs with each other using a mathematical method called a network meta‐analysis.

**What are the main results of our review?**

Compared to placebo, rivaroxaban added to antiplatelet medicines reduces all‐cause death and probably reduces cardiovascular death after heart attack. Dabigatran may reduce all‐cause death. Apixaban may provide no additional benefits compared with placebo in terms of all‐cause death or cardiovascular death. However, apixaban and rivaroxaban increase the risk of major bleeding compared with placebo. We found no clear difference between individual doses of NOACs for death or major bleeding. However, apixaban (combined dose) is probably less effective than rivaroxaban or dabigatran for preventing all‐cause death after a heart attack.

**What are the limitations of the evidence?**

We have little confidence in the evidence for dabigatran because the study recruited fewer than 2000 participants and the results are consistent with no effect as well as considerable benefit, considerable harm, or both. We are moderately confident in some of the evidence for apixaban and rivaroxaban because the results are consistent with no effect as well as considerable benefit or considerable harm.

**How up to date is this evidence?**

The evidence is current to September 2022.

## Summary of findings

**Summary of findings 1:** Non‐vitamin‐K‐antagonist oral anticoagulants versus placebo in adults with acute myocardial infarction and without an indication for anticoagulation: all‐cause mortality

**Patient or population:** adults after AMI without an indication for anticoagulation **Settings:** secondary care **Intervention:** NOACs (apixaban, rivaroxaban, dabigatran), all doses combined **Comparison:** placebo **Outcome:** all‐cause mortality								
**Comparison**	**No. of participants (no. of studies)**	**Direct evidence** **RR (95% CI)**	**Indirect evidence** **RR (95% CI)**	**NMA** **RR (95% CI)**	**Anticipated absolute effects estimate of the NMA**			**Certainty of the evidence of the NMA**
					**Risk with placebo**	**Risk with intervention**	**Risk difference with intervention**	
**Apixaban (all doses combined) vs placebo**	8638 (2)	**1.09** (0.88 to 1.35)	—	**1.09** (0.88 to 1.35)	36 per 1000	39 per 1000 (32 to 49)	3 more per 1000 (4 fewer to 13 more)	⊕⊕⊕⊝ **Moderate**^a^
**Rivaroxaban (all doses combined) vs placebo**	21,870 (3)	**0.82** (0.69 to 0.98)	—	**0.82** (0.69 to 0.98)	25 per 1000	20 per 1000 (17 to 24)	4 fewer per 1000 (8 fewer to 0 fewer)	⊕⊕⊕⊕ **High**
**Dabigatran (all doses combined) vs placebo**	1861 (1)	**0.57** (0.31 to 1.06)	—	**0.57** (0.31 to 1.06)	38 per 1000	22 per 1000 (12 to 40)	16 fewer per 1000 (26 fewer to 2 more)	⊕⊕⊝⊝ **Low**^b^
**AMI:** acute myocardial infarction; **CI:** confidence interval; **NMA:** network meta‐analysis; **NOAC:** non‐vitamin K antagonist oral anticoagulant; **RR:** risk ratio.								
**GRADE Working Group grades of evidence** **High certainty:** we are very confident that the true effect lies close to that of the estimate of the effect. **Moderate certainty:** we are moderately confident in the effect estimate; the true effect is likely to be close to the estimate of the effect, but there is a possibility that it is substantially different. **Low certainty:** our confidence in the effect estimate is limited; the true effect may be substantially different from the estimate of the effect. **Very low certainty:** we have very little confidence in the effect estimate; the true effect is likely to be substantially different from the estimate of effect.								

^a^ Downgraded one level for imprecision: 95% CI includes no effect and default value for appreciable harm (> 1.25). ^b^ Downgraded two levels for imprecision: 95% CI includes no effect and default value for appreciable benefit (< 0.75), or both, and the optimal information size was not met (i.e. sample size < 2000 participants).

**Summary of findings 2:** Non‐vitamin‐K‐antagonist oral anticoagulants versus placebo in adults with acute myocardial infarction and without an indication for anticoagulation: cardiovascular mortality

**Patient or population:** adults after AMI without an indication for anticoagulation **Settings:** secondary care **Intervention:** NOACs (apixaban, rivaroxaban, dabigatran), all doses combined **Comparison:** placebo **Outcome:** cardiovascular mortality								
**Comparison**	**No. of participants (no. of studies)**	**Direct evidence** **RR (95% CI)**	**Indirect evidence** **RR (95% CI)**	**NMA** **RR (95% CI)**	**Anticipated absolute effects estimate of the NMA**			**Certainty of the evidence of the NMA**
					**Risk with placebo**	**Risk with intervention**	**Risk difference with intervention**	
**Apixaban (all doses combined) vs placebo**	8638 (2)	**0.99** (0.77 to 1.27)	—	**0.99** (0.77 to 1.27)	28 per 1000	28 per 1000 (21 to 35)	0 fewer per 1000 (6 fewer to 8 more)	⊕⊕⊕⊝ **Moderate**^a^
**Rivaroxaban (all doses combined) vs placebo**	21,870 (3)	**0.83** (0.69 to 1.01)	—	**0.83** (0.69 to 1.01)	22 per 1000	18 per 1000 (15 to 22)	4 fewer per 1000 (7 fewer to 0 fewer)	⊕⊕⊕⊝ **Moderate**^a^
**Dabigatran (all doses combined) vs placebo**	1861 (1)	**0.72** (0.34 to 1.52)	—	**0.72** (0.34 to 1.52)	24 per 1000	17 per 1000 (8 to 37)	7 fewer per 1000 (16 fewer to 13 more)	⊕⊕⊝⊝ **Low**^b^
**AMI:** acute myocardial infarction; **CI:** confidence interval; **NMA:** network meta‐analysis; **NOAC:** non‐vitamin K antagonist oral anticoagulant; **RR:** risk ratio.								
**GRADE Working Group grades of evidence** **High certainty:** we are very confident that the true effect lies close to that of the estimate of the effect. **Moderate certainty:** we are moderately confident in the effect estimate; the true effect is likely to be close to the estimate of the effect, but there is a possibility that it is substantially different. **Low certainty:** our confidence in the effect estimate is limited; the true effect may be substantially different from the estimate of the effect. **Very low certainty:** we have very little confidence in the effect estimate; the true effect is likely to be substantially different from the estimate of effect.								

^a^ Downgraded one level for imprecision: 95% CI includes no effect and default value for appreciable harm (> 1.25) or appreciable benefit (< 0.75). ^b^ Downgraded two levels for imprecision: 95% CI includes no effect and default values for appreciable harm (> 1.25) and appreciable benefit (< 0.75), and the optimal information size was not met (i.e. sample size < 2000 participants).

**Summary of findings 3:** Non‐vitamin‐K‐antagonist oral anticoagulants versus placebo in adults with acute myocardial infarction and without an indication for anticoagulation: major bleeding

**Patient or population:** adults after AMI without an indication for anticoagulation **Settings:** secondary care **Intervention:** NOACs (apixaban, rivaroxaban, dabigatran) ‐ all doses combined **Comparison:** placebo **Outcome:** major bleeding								
**Comparison**	**No. of participants (no. of studies)**	**Direct evidence** **RR (95% CI)**	**Indirect evidence** **RR (95% CI)**	**NMA** **RR (95% CI)**	**Anticipated absolute effects estimate of the NMA**			**Certainty of the evidence of the NMA**
					**Risk with placebo**	**Risk with intervention**	**Risk difference with intervention**	
**Apixaban (all doses combined) vs placebo**	8544 (2)	**2.41** (1.44 to 4.06)	—	**2.41** (1.44 to 4.06)	5 per 1000	11 per 1000 (7 to 19)	7 more per 1000 (2 more to 14 more)	⊕⊕⊕⊕ **High**
**Rivaroxaban (all doses combined) vs placebo**	21,870 (3)	**3.31** (1.12 to 9.77)	—	**3.31** (1.12 to 9.77)	4 per 1000	12 per 1000 (4 to 35)	8 more per 1000 (0 fewer to 32 more)	⊕⊕⊕⊕ **High**
**Dabigatran (all doses combined) vs placebo**	1861 (1)	**1.74** (0.22 to 14.12)	—	**1.74** (0.22 to 14.12)	3 per 1000	5 per 1000 (1 to 38)	2 more per 1000 (2 fewer to 35 more)	⊕⊕⊝⊝ **Low**^a^
**AMI:** acute myocardial infarction; **CI:** confidence interval; **NMA:** network meta‐analysis; **NOAC:** non‐vitamin K antagonist oral anticoagulant; **RR:** risk ratio.								
**GRADE Working Group grades of evidence** **High certainty:** we are very confident that the true effect lies close to that of the estimate of the effect. **Moderate certainty:** we are moderately confident in the effect estimate; the true effect is likely to be close to the estimate of the effect, but there is a possibility that it is substantially different. **Low certainty:** our confidence in the effect estimate is limited; the true effect may be substantially different from the estimate of the effect. **Very low certainty:** we have very little confidence in the effect estimate; the true effect is likely to be substantially different from the estimate of effect.								

^a^ Downgraded two levels for imprecision: 95% CI includes no effect and default values for appreciable harm (> 1.25) and appreciable benefit (< 0.75), and the optimal information size was not met (i.e. sample size < 2000 participants).

## Background

### Description of the condition

Acute myocardial infarction (AMI) is the death of the myocardial tissue due to ischaemia. AMI occurs secondary to an obstruction in one or more coronary arteries due to a rupture of an atherosclerotic plaque. AMI is divided into ST‐segment elevation myocardial infarction (STEMI) and non‐ST‐segment elevation myocardial infarction (NSTEMI), according to the electrocardiographic appearance of the lesion.

Despite therapy, AMI remains a life‐threatening disease: up to one in five affected people either die, suffer recurrent myocardial infarction, or develop a stroke within one year (Jernberg 2015). The estimated global incidence of AMI is 10 to 15 million episodes per year (James 2018; Vos 2016). AMI has a considerable economic burden: in the USA, hospitalisation due to AMI costs USD 14.3 billion each year (Liang 2020), while the annual medical costs of ischaemic heart disease in Europe are estimated at EUR 59 billion (Wilkins 2017). The economic burden associated with AMI in China is higher than in some high‐income economies (Jan 2018).

The prognosis of AMI has improved markedly since the early 2000s because of advancements in treatment strategies (Ibanez 2018; Roffi 2016). One key contributor to improved outcomes is dual antiplatelet therapy (DAPT) with aspirin and a P2Y12 receptor antagonist. DAPT has significantly reduced the risk of recurrent cardiovascular events, including stent thrombosis, particularly in people undergoing percutaneous coronary intervention (PCI; Leon 1998; Valgimigli 2017).

While DAPT reduces the incidence of stent thrombosis in the first few months after PCI, the impact of DAPT on late and particularly very late stent thrombosis is less certain (Garg 2015). Generally, the recommended strategy after AMI is DAPT for at least 12 months, followed by life‐long single antiplatelet therapy (SAPT; Amsterdam 2014; Collet 2020; Ibanez 2018; O'Gara 2013; Roffi 2016). However, DAPT duration can be shortened or lengthened (beyond 12 months) according to each person's ischaemic or bleeding risk profile (Bonaca 2015; Kikkert 2018).

The potent P2Y12 inhibitors prasugrel and ticagrelor are favoured over clopidogrel for DAPT without anticoagulation following AMI (Collet 2020; Ibanez 2018; Roffi 2016). However, even with DAPT, recurrent ischaemic events remain high (Al Said 2018), owing to excessive thrombin generation and adverse fibrin clots that resist lysis (Merlini 1994; Sumaya 2018a). Additional anticoagulation on top of DAPT may limit adverse fibrin properties (Sumaya 2018b; Varin 2013). Researchers have further evaluated this finding in clinical studies combining non‐vitamin‐K‐antagonist oral anticoagulants (NOACs) with antiplatelet therapy in AMI management.

### Description of the intervention

NOACs, also known as direct‐acting oral anticoagulants (DOACs), have been developed as an alternative to vitamin K antagonists (VKAs) such as warfarin. While VKAs reduce the synthesis of functional vitamin K‐depending clotting factors II, VII, IX, and X, and proteins C and S, NOACs directly inhibit an activated clotting factor (factor IIa or factor Xa). Four NOACs are currently approved for clinical use: dabigatran, which is a thrombin inhibitor; and rivaroxaban, apixaban, and edoxaban, which are direct factor Xa inhibitors (Bauer 2013).

NOACs are usually well tolerated and cause few side effects. However, unlike VKAs, NOACs cannot be easily reversed in major bleeding. NOAC reversal agents, such as idarucizumab and andexanet alfa, can help treat people with life‐threatening bleeding or those needing immediate surgery (Cuker 2019; Glund 2015; Pollack 2015). Other disadvantages of NOACs compared to VKAs include their higher price and the absence of laboratory testing to objectively determine compliance. Moreover, dose adjustments of NOACs are necessary for people with renal impairment or with low or very high weight (Al Said 2019). The advantages of NOACs include a rapid onset of action without the need for regular monitoring or perioperative bridging with parenteral anticoagulants (Bauer 2013; Eriksson 2011). NOACs are at least as effective as warfarin in preventing stroke in non‐valvular atrial fibrillation (Connolly 2009; Giugliano 2013; Granger 2011; Patel 2011). However, the key advantage is the safer profile of NOACs: compared with warfarin, they cause less major bleeding, particularly intracranial haemorrhage (Connolly 2009; Giugliano 2013; Granger 2011; Patel 2011). Moreover, compared with VKAs, NOACs may be safer and equally effective in people with an indication for anticoagulation due to non‐valvular atrial fibrillation (Al Said 2019).

These safety and efficacy considerations have led to the exploration of NOACs in secondary prevention after AMI. Studies have found that VKAs alone, or in combination with aspirin, reduce rates of major adverse cardiovascular events (MACEs) but increase the rate of major bleeding, including intracranial haemorrhage (Anand 2003; Andreotti 2006; Hurlen 2002; Rothberg 2005; van Es 2002). The antithrombotic potential of NOACs after AMI in people without an indication for anticoagulation remains unclear.

Several studies have assessed the efficacy of NOACs combined with DAPT after acute coronary syndrome (ACS; Alexander 2009; Gibson 2011; Mega 2012; Oldgren 2011). Dabigatran and apixaban showed no significant clinical benefit in preventing new ischaemic cardiovascular events (Alexander 2009; Oldgren 2011). Moreover, apixaban was prematurely discontinued due to a significant increase in the risk of major bleeding events (Alexander 2011). On the other hand, a very low dose of rivaroxaban (2.5 mg twice daily (BD)) resulted in reduced MACEs after ACS (Gibson 2011; Mega 2012).

### How the intervention might work

NOACs inhibit thrombin either directly (dabigatran) or indirectly by inhibiting factor Xa (rivaroxaban, apixaban, and edoxaban). AMIs lead to increased thrombin generation, and elevated thrombin concentrations are detectable for at least six months following the acute episode (Merlini 1994). Furthermore, elevated thrombin levels are linked to the recurrence of cardiovascular events. Multiple studies have demonstrated the importance of coagulation's protein arm, represented by the ability to lyse fibrin, in recurrent events following ACS (Farag 2019; Saraf 2010; Sumaya 2018a; Sumaya 2020). These studies indicate a significant role of thrombin generation in arterial thrombosis. NOACs may improve outcomes by limiting arterial thrombosis through their ability to inhibit thrombin formation. Furthermore, anticoagulation promotes fibrin clot lysis (Sumaya 2018a), which enhances reperfusion following a plaque rupture event. Anticoagulants also exert an indirect antiplatelet effect by inhibiting thrombin generation (Sumaya 2018a).

### Why it is important to do this review

Balancing the risk of bleeding and thrombosis after AMI is challenging, and the optimal antithrombotic therapy remains uncertain. The role of NOACs after AMI is not fully understood, and treatment decisions rely on limited evidence. Current European guidelines provide a class IIb recommendation (usefulness/efficacy is less well established by evidence/opinion) for considering the use of rivaroxaban 2.5 mg BD, in combination with aspirin and clopidogrel, for people with NSTEMI who have high ischaemic and low bleeding risks (Collet 2020; Roffi 2016). Low‐dose rivaroxaban may be suitable for selected people with low bleeding risk who receive aspirin and clopidogrel after STEMI (class IIb recommendation; Ibanez 2018). The National Institute for Health and Care Excellence (NICE) has approved rivaroxaban with either aspirin alone or aspirin plus clopidogrel as an option to avoid additional blood clots after ACS in people with high ischaemic risk (NICE 2015). NOACs have not been approved for ACS treatment in the USA and are therefore not recommended in the STEMI or NSTEMI guidelines (Amsterdam 2014; O'Gara 2013).

This systematic review aims to assess the evidence for the safety and efficacy of NOACs after AMI to help establish the optimal level of anticoagulation and identify the patient group with the most favourable balance of benefit and risk associated with NOACs in combination with antiplatelets (Figure 1, Figure 2, Figure 3).

**1:**
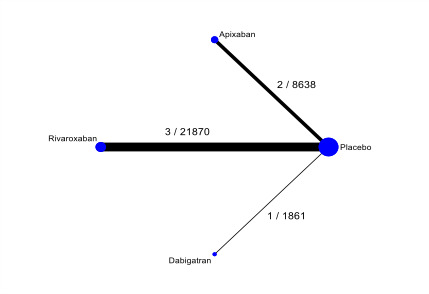
Network diagram for primary outcomes ‐ primary analyses (non‐vitamin‐K‐antagonist oral anticoagulants, all doses combined): all‐cause death and cardiovascular death (Primary outcomes). Circles represent the drug as a node in the network; lines represent direct comparisons. Nodes are weighted according to the number of studies that included the respective intervention. Edges are weighted according to the number of participants included in the respective comparison. Numbers on the lines represent the number of trials and participants for each comparison. We combined these two primary outcomes in a single plot since they have the same number of interventions, studies, and participants.

**2:**
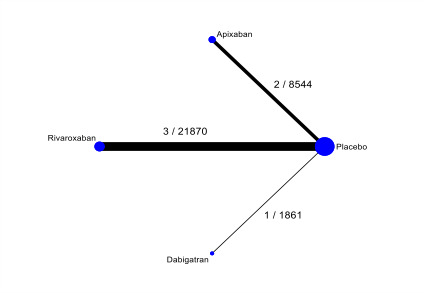
Network diagram for primary outcomes ‐ primary analyses (non‐vitamin‐K‐antagonist oral anticoagulants, all doses combined): major bleeding (Primary outcomes). Circles represent the drug as a node in the network. Lines represent direct comparisons. Nodes are weighted according to the number of studies that included the respective intervention. Edges are weighted according to the number of participants included in the respective comparison. Numbers on the lines represent the number of trials and participants for each comparison.

**3:**
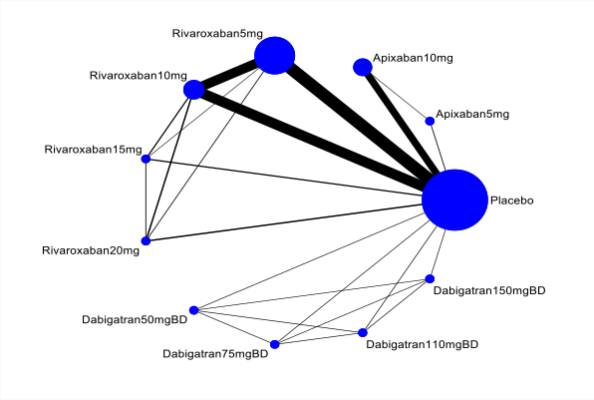
Network plot for primary outcomes ‐ secondary analyses (differences doses of non‐vitamin‐K‐antagonist oral anticoagulants): all‐cause death, cardiovascular death, and major bleeding (Primary outcomes). Circles represent the drug as a node in the network. Lines represent direct comparisons. Nodes are weighted according to the number of studies that included the respective intervention. Edges are weighted according to the number of participants included in the respective comparison. We combined these secondary outcomes in a single plot since they have the same number of interventions, studies, and participants.

Given the complexity of the condition and the absence of randomised controlled trials (RCTs) comparing different NOACs against each other, it is essential to carry out a comprehensive and comparative evaluation of all available treatment options within a network meta‐analysis (NMA) framework. At the time of writing, there were no other published systematic reviews and NMAs assessing the efficacy and safety of NOACs after AMI. We therefore aimed to present the most current evidence for the use of patients, clinicians, policymakers, and researchers.

## Objectives

To evaluate the efficacy and safety of non‐vitamin‐K‐antagonist oral anticoagulants (NOACs) in addition to background antiplatelet therapy, compared with placebo, antiplatelet therapy, or both, after acute myocardial infarction (AMI) in people without an indication for anticoagulation (i.e. atrial fibrillation or venous thromboembolism).

## Methods

### Criteria for considering studies for this review

#### Types of studies

Parallel‐arm RCTs with individual or cluster randomisation were eligible for inclusion. We excluded cross‐over trials as the different treatment alternatives can mutually affect each other and potentially contaminate the analysis. Because the interventions have a long elimination half‐life, a carry‐over effect is likely. Moreover, our outcomes of interest are either irreversible (such as mortality) or of long duration.

#### Types of participants

We included adults (aged 18 years or older) with an AMI (NSTEMI or STEMI) and without an indication for oral anticoagulation. We excluded participants with the following comorbidities/characteristics.

- Active bleeding or high bleeding risk
- Known coagulopathy
- Previous intracranial haemorrhage, ischaemic stroke, or transient ischaemic attack
- Severe renal dysfunction with a calculated creatinine clearance of less than 20 mL/minute
- A severe comorbid condition with a life expectancy of six months or less
- Pregnancy, breastfeeding, or, in women of childbearing potential, inability to use an acceptable method of contraception

In trials with mixed populations (i.e. where only some participants met the eligibility criteria), we included only the eligible participants if their data were reported separately or could be obtained from trial authors. Otherwise, we included studies with a mixed population if more than 50% of the participants met the eligibility criteria.

#### Types of interventions

We were interested in the following experimental interventions.

- Dabigatran‐based therapy (i.e. dabigatran in combination with SAPT or DAPT)
- Rivaroxaban‐based therapy (i.e. rivaroxaban in combination with SAPT or DAPT)
- Apixaban‐based therapy (i.e. apixaban in combination with SAPT or DAPT)
- Edoxaban‐based therapy (i.e. edoxaban in combination with SAPT or DAPT)

Eligible controls were placebo, an antiplatelet‐based antithrombotic strategy (SAPT/DAPT), or both.

We included trials comparing any type of NOAC (dabigatran, rivaroxaban, apixaban, edoxaban) with control, and head‐to head trials of different NOACs.

Our assessment involved both direct and indirect comparisons. For direct comparisons, we investigated the efficacy and safety of each individual NOAC when compared to placebo. For indirect comparisons, we explored how NOACs (dabigatran, rivaroxaban, apixaban, edoxaban) performed relative to one another.

We excluded NOACs that were not licenced by the US Food and Drug Administration (FDA) or European Medicines Agency (EMA) due to lack of safety or effectiveness (e.g. betrixaban, darexaban, eribaxaban, letaxaban, nokxaban, AZD‐0837, fidexaban, LY517717, odiparcil, otamixaban, TTP889, and ximelagatran), as they were not clinically relevant. We assumed that people who fulfilled the inclusion criteria were equally eligible to be randomised to any of the interventions we planned to compare.

#### Types of outcome measures

Reporting one or more of the outcomes listed below in the trial was not an inclusion criterion for the review. Where a published study did not report one of these outcomes, we accessed the trial protocol and contacted the trial authors to ascertain whether the outcome was measured but not reported. For the outcomes that could occur more than once in a participant during the trial, we measured the number of participants with at least one event.

##### Primary outcomes

- All‐cause mortality
- Cardiovascular mortality
- Major bleeding

##### Secondary outcomes

- Myocardial infarction
- Stroke (ischaemic, haemorrhagic, or of uncertain cause)
- Stent thrombosis
- Non‐major bleeding
- Recurrent hospitalisation
- Systemic embolism
- Health‐related quality of life, assessed using validated instruments (e.g. 36‐Item Short‐Form Health Survey (SF‐36), EuroQol Five‐Dimension Health Survey (EQ‐5D))

We assessed all outcomes at the longest point of follow‐up for each trial. We accepted the definitions of clinical event outcomes (e.g. stroke, myocardial infarction) provided in the individual trials. We defined major bleeding according to the Thrombolysis In Myocardial Infarction (TIMI) criteria (Chesebro 1987; Mehran 2011). Non‐major bleeding was any bleeding that did not fit the TIMI major bleeding criteria. We defined stent thrombosis according to the Academic Research Consortium (ARC) criteria (Cutlip 2007). Recurrent hospitalisation was a dichotomous outcome (more than one hospitalisation after randomisation and during follow‐up, yes/no).

### Search methods for identification of studies

#### Electronic searches

We identified trials through systematic searches of the following bibliographic databases.

- Cochrane Central Register of Controlled Trials (CENTRAL) in the Cochrane Library (Issue 9 of 12, 2022)
- MEDLINE ALL (Ovid, 1946 to 22 September 2022)
- Embase (Ovid, 1980 to 2022, week 37)
- Conference Proceedings Citation Index – Science (CPCI‐S) on Web of Science (Clarivate Analytics, 1990 to 23 September 2022)

Appendix 1 shows our preliminary search strategy for MEDLINE (Ovid). We applied the Cochrane sensitivity‐maximising RCT filter to the MEDLINE strategy and adapted it to the other databases, except CENTRAL (Lefebvre 2022). We also searched ClinicalTrials.gov (www.clinicaltrials.gov) and the World Health Organization (WHO) International Clinical Trials Registry Platform (ICTRP; trialsearch.who.int) for ongoing or unpublished trials on 23 September 2022.

We searched all databases from their inception and imposed no restrictions on language or status of publication. We did not perform a separate search for the adverse effects of NOACs, considering only those described in the included studies.

#### Searching other resources

We checked the reference lists of all included studies and any relevant systematic reviews for additional references to trials. We also examined any relevant errata and retraction statements related to included studies.

### Data collection and analysis

#### Selection of studies

Two review authors (SAS, SA) independently screened the titles and abstracts of all the records identified in the search and coded them as 'retrieve' (eligible or potentially eligible/unclear) or 'do not retrieve'. A third review author (WS) arbitrated if any disagreements arose. We retrieved the full‐text study reports/publications of eligible and potentially eligible/unclear studies. Two review authors (SAS, SA) independently screened the full texts and identified studies for inclusion. They also identified and recorded reasons for exclusion of the ineligible studies. We resolved any disagreement through discussion or, if required, by consulting a third review author (WS). We identified and excluded duplicates and collated multiple reports of the same study so that each study, rather than each report, was the unit of interest in the review. We recorded the selection process in sufficient detail to complete a Characteristics of excluded studies table and a PRISMA flow diagram (Page 2021).

#### Data extraction and management

Two review authors (SAS, SA) independently extracted data from the included trials. We extracted and collated the following data using a standardised data extraction form.

- Methods: study design, total duration of study, details of any run‐in period, number of study centres and location, study setting, date of study
- Participants: number randomised, number lost to follow‐up/withdrawn, number analysed, mean age, age range, sex, inclusion criteria, exclusion criteria, type of myocardial infarction (NSTEMI, STEMI), kidney function
- Interventions: intervention, doses of the intervention**,** comparison, concomitant medications, excluded medications
- Outcomes: primary and secondary outcomes specified and collected, time points reported, number of participants with the events and total number of participants randomised for dichotomous outcomes, and relative treatment effects (e.g. risk ratio (RR)) with relative 95% confidence interval (CI)
- Notes: funding for trial, notable conflicts of interest of trial authors

From each study, we extracted the following potential effect modifiers: age, sex, lipid levels, body mass index (BMI), comorbidities and embolic risk. Two review authors (SAS, SA) independently extracted the outcome data from the included studies. We resolved any disagreements by consensus or by involving a third review author (WS), if necessary. One review author (SA) transferred the data to Review Manager Web (Review Manager 2020). We double‐checked correct data entry by comparing the data presented in the systematic review with the data extraction form. A second review author (SAS) spot‐checked study characteristics for accuracy against the trial reports.

#### Assessment of risk of bias in included studies

Two review authors (SAS, SA) independently assessed the risk of bias for each trial using the criteria outlined in the *Cochrane Handbook for Systematic Reviews of Interventions* (Higgins 2017). We resolved any disagreements by discussion or by involving another review author (WS). We assessed the risk of bias according to the following domains.

- Random sequence generation
- Allocation concealment
- Blinding of participants and personnel
- Blinding of outcome assessment
- Incomplete outcome data
- Selective outcome reporting
- Other bias

Had we identified any eligible cluster‐RCTs, we would have considered the following additional risk of bias domains for those trials.

- Recruitment bias
- Baseline imbalance
- Loss of clusters
- Incorrect analysis
- Comparability with individually randomised trials

We graded each trial as being at high, low, or unclear risk of bias for each domain. We provided a quote from the study report, together with a justification for our judgement, in the risk of bias section of the Characteristics of included studies table. We summarised the risk of bias judgements across different studies for each of the domains listed. Where information on risk of bias related to unpublished data or correspondence with a trialist, we noted this in the risk of bias section. When examining treatment effects, we considered the risk of bias for the studies that contributed to that outcome.

#### Measures of treatment effect

We analysed dichotomous data using risk ratios (RRs) with 95% confidence intervals (CIs). For continuous data, we planned to use mean differences (MDs) with 95% CIs where different studies measured the outcome on the same scale. If we had identified studies that used different scales to measure the same continuous outcome, we would have used the standardised mean difference (SMD). We would have interpreted SMDs using generic effect size estimates, as follows (Cohen 1998).

- Small/minor SMD: 0.2 or less
- Medium SMD: 0.2 to 0.8
- Large SMD: 0.8 or greater

We did not include time‐to‐event data but did include dichotomous data at different time points.

We calculated the NNTB (number needed to treat for an additional beneficial outcome) or NNTH (number needed to treat for an additional harmful outcome) values from the RR according to the formula NNTB or NNTH = 1/ACR*(1−RR), where ACR is the assumed control risk (Higgins 2019).

#### Unit of analysis issues

Our unit of analysis was the participant. If trials compared more than two interventions that were eligible for inclusion in this review, we divided the participants in the control group into two or more groups for the pairwise meta‐analysis; in this way, we avoided double‐counting participants in the control group. We presented the longest point of follow‐up for each trial. We treated multiarm studies as multiple independent comparisons in pairwise meta‐analyses.

For the NMA, we accounted for the correlation between the effect sizes from multiarm studies using the approach suggested by Rücker and Schwarzer, which utilises back‐calculated standard errors in the weighted least‐square estimator to reflect the within‐study correlation (Rücker 2012; Rücker 2014; Rücker 2015).

Cross‐over trials were not eligible for inclusion, and we identified no eligible cluster‐randomised trials.

#### Dealing with missing data

We contacted investigators or study sponsors to obtain missing numerical outcome data where possible. We obtained very few unpublished data on all individual doses of rivaroxaban from one phase II trial (ATLAS ACS). In the case of missing statistics (such as standard deviations), we had intended to contact the trial authors; however, this was not necessary.

#### Assessment of heterogeneity

In the pairwise meta‐analyses, we assessed heterogeneity by visually inspecting the forest plots. We quantified heterogeneity using the I² statistic, interpreting the values according to the following thresholds, as recommended in the *Cochrane Handbook for Systematic Reviews of Interventions* (Deeks 2022).

- 0% to 40%: might not be important.
- 30% to 60%: may represent moderate heterogeneity.
- 50% to 90%: may represent substantial heterogeneity.
- 75% to 100%: represents considerable heterogeneity.

In the NMAs, we evaluated coherence, which is the statistical manifestation of the transitivity assumption. Transitivity refers to the assumption that the distribution of effect modifiers is balanced across treatment comparisons.

In the case of relevant incoherence in the NMAs for the primary outcomes, we had planned to explore possible sources and conduct subgroup and sensitivity analyses based on factors described in the Subgroup analysis and investigation of heterogeneity and Sensitivity analysis sections.

The link between transitivity and coherence is a critical aspect of the NMA. Transitivity, in the context of NMA, refers to the assumption that the distribution of effect modifiers is balanced across treatment comparisons. Coherence, on the other hand, is the statistical representation of transitivity, reflecting the agreement between the network's direct and indirect comparisons. Incoherence indicates possible violations of the transitivity assumption or other causes of bias (Chaimani 2022). To assess for local inconsistency, we employed the node‐splitting approach using the 'netsplit' command of the 'netmeta' R package, which allowed us to separate network estimates into the contributions of direct and indirect evidence (Rücker 2017). Unfortunately, we were unable to create net heat plots due to the limited number of included studies (Jackson 2012; Krahn 2013).

#### Assessment of intransitivity across treatment comparisons

We considered transitivity by assessing clinical and methodological comparability. Given the similar inclusion criteria and comparable included populations in the various RCTs, we considered the transitivity assumption withstanding, assuming the following.

- The common treatment used to compare different NOACs indirectly was similar in the different trials.
- No relevant variation in effect modifiers (age, sex, lipid levels, BMI, comorbidities, and embolic risk) was identified between trials.

#### Assessment of reporting biases

We sought to examine the risk of publication bias in our NMA by visually inspecting funnel plots for each direct comparison (edge) in the network. We would have examined funnel plots for any asymmetry, which could suggest publication bias or other reporting biases. However, due to the small number of studies in our network, we were unable to conduct a detailed analysis of small‐study effects using funnel plots.

#### Data synthesis

##### Methods for direct treatment comparisons

We conducted pairwise meta‐analyses using random‐effects models in Review Manager Web (Review Manager 2020) for every treatment comparison with at least two studies. With a random‐effects model, the true effect size may or may not vary from study to study, and the model does not assume that either is the case. As part of the analysis, the amount of variance in true effects is estimated across studies, and the estimate may or may not be zero. With a fixed‐effect model, the true effect size does not vary from study to study. Therefore, the fixed‐effect model is more restrictive: it imposes a constraint that is neither necessary nor plausible.

##### Methods for indirect and mixed treatment comparisons

To evaluate the feasibility of NMA, we conducted a thorough examination of the network diagrams' geometry. This assessment involved scrutinising the structure of the network to determine its suitability for NMA. Specifically, we analysed the relationships between different treatments to ensure that the network possessed adequate evidence for meaningful treatment comparisons.

Our evaluation focused on two key criteria: network connectivity and the sufficiency of information. A connected network (indicating relationships between treatments) and a substantial amount of evidence within the network are essential for meaningful NMA. When we refer to 'sufficiency of information,' we mean having a sufficient quantity and quality of data within the network of studies. This includes an adequate number of studies and participants for each treatment comparison and overall study quality.

If these criteria were met, we proceeded with NMA; otherwise, we opted for pairwise meta‐analyses. Where the evidence was suitable for NMA, we performed a multivariate random‐effects meta‐analysis of the primary outcomes within a frequentist framework using the R package 'netmeta' (Rücker 2017). This technique allows for the inclusion of multiarm studies. We planned to perform the analyses by considering treatments collapsed according to doses and by considering different doses of the same treatment as single nodes in the network.

We performed NMAs for all primary outcomes at the latest point of follow‐up for each trial (Primary outcomes): the primary analysis involved NMA where treatments with different doses were combined, and the secondary analysis involved NMA where the treatments were split according to dose.

The nodes of the network are the interventions specified in the review inclusion criteria; we did not combine any. We added a network plot for each primary outcome (Figure 1; Figure 2; Figure 3).

We presented all results as summary relative effects (RRs) for each possible pair of treatments. We estimated the relative rankings for the primary outcomes using P scores, which are derived from the P values of all pairwise comparisons and enable ranking of treatments on a continuous 0‐to‐1 scale. P scores are based solely on the point estimates and standard errors of the frequentist NMA estimates under the normality assumption. P scores measure the mean extent of certainty that a treatment is better than the competing treatments (Rücker 2015). Larger P scores indicate a higher ranking of the included treatment. This interpretation is comparable to that of the surface under the cumulative ranking curve (SUCRA; Rücker 2015).

###### League table

We created league tables using the primary outcomes (all‐cause mortality, cardiovascular mortality, and major bleeding). League tables use a matrix structure, where the upper triangle presents the results from direct (pairwise) meta‐analyses, and the lower triangle presents the results from the NMAs (Chaimani 2022). Comparisons between treatments are read from left to right; the estimate is in the cell in common between the column‐defining treatment and the row‐defining treatment. We presented results as RRs (95% CIs), where an RR below 1 favours the row‐defining treatment (Table 4; Table 5; Table 6).

**1:** League table – all‐cause mortality

**Pairwise meta‐analysis**			
**Placebo**	1.09 (0.88 to 1.35)	0.82 (0.69 to 0.98)	0.57 (0.31 to 1.06)
1.09 (0.88 to 1.35)	**Apixaban**	—	—
0.82 (0.69 to 0.98)	1.33 (1.01 to 1.76)	**Rivaroxaban**	—
0.57 (0.31 to 1.06)	1.92 (1.00 to 3.70)	1.45 (0.76 to 2.75)	**Dabigatran**
**Network meta‐analysis**			

Comparisons between treatments should be read from left to right, and the estimate is in the cell in common between the column‐defining treatment and the row‐defining treatment. The upper triangle presents the results from direct (pairwise) meta‐analyses, and the lower triangle presents the results from the NMA. The lower triangle contains the RRs of the NMA (mixed) effect estimates comparing the treatment in the row versus the treatment in the column, whereas cells in the upper triangle refer to the RR direct effect estimates comparing the treatment in the column versus the treatment in the row. If the (mixed or direct) RR for A versus B is available, the B versus A comparison can be easily calculated as 1/RR (the inverse of the estimated effect). Results are presented as RR (95% CI), where an RR < 1 favours the row‐defining treatment. The order of treatments in the diagonal is arbitrary and does not reflect ranking. CI: confidence interval; NMA: network meta‐analysis; RR: risk ratio.

**2:** League table – cardiovascular mortality

**Pairwise meta‐analysis**			
**Placebo**	0.99 (0.77 to 1.27)	0.83 (0.69 to 1.01)	0.72 (0.34 to 1.52)
0.99 (0.77 to 1.27)	**Apixaban**	—	—
0.83 (0.69 to 1.01)	1.19 (0.87 to 1.62)	**Rivaroxaban**	—
0.72 (0.34 to 1.52)	1.38 (0.63 to 3.03)	1.16 (0.54 to 2.51)	**Dabigatran**
**Network meta‐analysis**			

Comparisons between treatments should be read from left to right, and the estimate is in the cell in common between the column‐defining treatment and the row‐defining treatment. The upper triangle presents the results from direct (pairwise) meta‐analyses, and the lower triangle presents the results from the NMA. The lower triangle contains the RRs of the NMA (mixed) effect estimates comparing the treatment in the row versus the treatment in the column, whereas cells in the upper triangle refer to the RR direct effect estimates comparing the treatment in the column versus the treatment in the row. If the (mixed or direct) RR for A versus B is available, the B versus A comparison can be easily calculated as 1/RR (the inverse of the estimated effect). Results are presented as RR (95% CI), where an RR < 1 favours the row‐defining treatment. The order of treatments in the diagonal is arbitrary and does not reflect ranking. CI: confidence interval; NMA: network meta‐analysis; RR: risk ratio.

**3:** League table – major bleeding

**Pairwise meta‐analysis**			
**Placebo**	2.41 (1.44 to 4.06)	3.31 (1.12 to 9.77)	1.74 (0.22 to 14.12)
2.41 (1.44 to 4.06)	**Apixaban**	—	—
3.31 (1.12 to 9.77)	0.67 (0.15 to 2.94)	**Rivaroxaban**	—
1.74 (0.22 to 14.12)	1.24 (0.08 to 18.21)	1.84 (0.14 to 24.75)	**Dabigatran**
**Network meta‐analysis**			

Comparisons between treatments should be read from left to right, and the estimate is in the cell in common between the column‐defining treatment and the row‐defining treatment. The upper triangle presents the results from direct (pairwise) meta‐analyses, and the lower triangle presents the results from the NMA. The lower triangle contains the RRs of the NMA (mixed) effect estimates comparing the treatment in the row versus the treatment in the column, whereas cells in the upper triangle refer to the RR direct effect estimates comparing the treatment in the column versus the treatment in the row. If the (mixed or direct) RR for A versus B is available, the B versus A comparison can be easily calculated as 1/RR (the inverse of the estimated effect). Results are presented as RR (95% CI), where an RR < 1 favours the row‐defining treatment. The order of treatments in the diagonal is arbitrary and does not reflect ranking. CI: confidence interval; NMA: network meta‐analysis; RR: risk ratio.

#### Subgroup analysis and investigation of heterogeneity

We planned to carry out the following subgroup analyses for primary and secondary outcomes where we identified substantial heterogeneity.

- Type of myocardial infarction: NSTEMI versus STEMI
- Mean age of participants in each trial: 75 years and older versus younger than 75 years
- People with mild versus moderate kidney dysfunction as determined at screening according to the Cockcroft‐Gault formula (mild impairment: creatinine clearance 60 mL/minute to < 90 mL/minute; moderate impairment: creatinine clearance 30 mL/minute to < 60 mL/minute)
- People with the usual full dose of NOAC versus reduced or adjusted dose
- Type of coronary stents: dual therapy stent, bioresorbable vascular scaffold, bio‐engineered stent, drug‐eluting stent, bare‐metal stent
- Evaluation of the involved coronary vessel (left main coronary artery, left anterior descending artery, left circumflex artery, right coronary artery)
- Concomitant use of antiplatelet therapy (DAPT versus SAPT; aspirin versus clopidogrel versus ticagrolor versus prasugrel)
- Funding status (studies with industry funding versus studies without industry funding)

Owing to the limited number of included studies, we were unable to investigate heterogeneity through subgroup analysis.

#### Sensitivity analysis

We planned to conduct a sensitivity analysis of our primary outcomes to assess the effect of excluding studies judged at unclear or high risk of bias in any domain. This was not possible, as all included studies were at low risk of bias in all domains.

#### Summary of findings and assessment of the certainty of the evidence

We created a summary of findings table using the NMA results of the comparison 'NOACs (all doses) versus placebo' for the primary outcomes: all‐cause mortality, cardiovascular mortality, and major bleeding (Primary outcomes). We used the five GRADE considerations (study limitations, consistency of effect, imprecision, indirectness, and publication bias) to assess the certainty of the body of evidence as it related to the studies that contributed data to the meta‐analyses for the prespecified primary outcomes. We also applied the four‐step approach presented by Brignardello‐Petersen and colleagues to rate the certainty of evidence in the NMA estimates (Brignardello‐Petersen 2020).

We used methods and recommendations described in the *Cochrane Handbook for Systematic Reviews of Interventions* (Schünemann 2022), employing GRADEpro GDT software (GRADEpro GDT 2015).

Two review authors (SAS, SA) independently judged the certainty of the evidence, resolving any disagreements by discussion or by involving a third review author (WS), if necessary. Judgements were justified, documented, and incorporated into the reporting of results for each outcome. We extracted study data, formatted our comparisons in data tables, and prepared a summary of findings table before writing the results and conclusions of our review.

## Results

### Description of studies

#### Results of the search

The literature search identified 2923 records, of which 876 were duplicates. We screened 2047 titles and abstracts and identified 73 records for full‐text assessment. Of these full‐text references, we included 53 and excluded 19. We also identified one ongoing trial (VaLiDate‐R; NCT03775746; see Characteristics of ongoing studies). The 53 references reported findings of six completed studies (Characteristics of included studies); we included all six completed studies in the meta‐analysis (APPRAISE 1; APPRAISE 2; ATLAS ACS; ATLAS ACS 2; GEMINI‐ACS; REDEEM). See Figure 4 for details.

**4:**
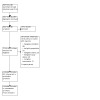
PRISMA study flow diagram.

#### Included studies

The Characteristics of included studies table and Table 7 provide detailed characteristics of the six included studies. All included trials were international multicentre trials. Follow‐up ranged from six to 13 months. Four trials were phase II RCTs (APPRAISE 1; ATLAS ACS; GEMINI‐ACS; REDEEM), and two were phase III RCTs (APPRAISE 2; ATLAS ACS 2). The included trials randomised a total of 33,039 participants, of whom 1715 were from APPRAISE 1, 7392 from APPRAISE 2, 3491 from ATLAS ACS, 15,526 from ATLAS ACS 2, 3037 from GEMINI‐ACS, and 1878 from REDEEM. All trials had more male participants (between 67% and 78%). The mean age ranged from 57 to 67 years.

**4:** Baseline characteristics of included trials

**Variable**	**APPRAISE 1**	**APPRAISE 2**	**ATLAS ACS**	**ATLAS ACS 2**	**GEMINI‐ACS**	**REDEEM**
**Design**	RCT (phase II)	RCT (phase III)	RCT (phase II)	RCT (phase III)	RCT (phase II)	RCT (phase II)
**Overall study population**	1715	7392	3491	15526	3037	1878
**NOAC type**	Apixaban	Apixaban	Rivaroxaban	Rivaroxaban	Rivaroxaban	Dabigatran
**NOAC dosages**	2.5 mg BD, 10 mg QD	5 mg BD	5 mg QD, 10 mg QD, 15 mg QD, 20 mg QD	2.5 mg BD, 5 mg BD	2.5 mg BD	50 mg BD, 75 mg BD, 110 mg BD, 150 mg BD
**Concomitant antiplatelet therapy**	All participants received aspirin, and 76% received additional clopidogrel.	All participants received aspirin, and 81% received additional clopidogrel.	All participants received aspirin and 80% received additional clopidogrel.	All participants received aspirin, and 93% received additional clopidogrel.	All participants received SAPT with either clopidogrel (43.9%) or ticagrelor (56.1%).	All participants received aspirin, and 93% received additional clopidogrel.
**Date of study**	May 2006–Oct 2007	Mar 2009–Nov 2010	Nov 2006–Oct 2008	Nov 2008–Sep 2011	Apr 2015–Oct 2016	Mar 2008–Oct 2009
**Follow‐up (months)**	6	8	6	13	11	6
**Centres/countries**	151/14	858/39	297/27	766/44	371/21	161/24
**N randomised**	1715	7392	3491	15,526	3037	1878
**Days to randomisation**	4	6	4	5	5	7
**Median age (years)**	61	67	57	62	62	62
**Age (> 65 years), %**	NR	59	24	37	42	45
**Sex (male), %**	77	67	78	75	75	75
**STEMI, %**	63	40	52	50	49	60
**NSTEMI, %**	28	41	30	26	40	40
**Unstable angina, %**	9	18	18	24	11	NR
**PCI for MI, %**	66	44	61	63	87	55
**Previous MI, %**	6	25	21	27	21	29
**Diabetes, %**	22	48	19	32	29	32
**Hypertension, %**	NR	NR	57	68	71	67
**Dyslipidaemia, %**	NR	NR	44	49	56	NR
**Smoker, %**	NR	12	62	NR	32	61
**Heart failure, %**	13	28	NR	NR	10	11
**Peripheral artery disease, %**	6	18	NR	NR	4	7
**Cerebrovascular disease, %**	4	10	NR	3	NR	NR
**Renal insufficiency, %**	29	28	NR	NR	NR	NR

BD: twice daily; MI: myocardial infarction; N: number; NOAC: non‐vitamin K antagonist oral anticoagulant; NR: not reported; NSTEMI: non‐ST‐segment elevation myocardial infarction; PCI: percutaneous coronary intervention; QD: once daily; RCT: randomised controlled trial; SAPT: single antiplatelet therapy; STEMI: ST‐segment elevation myocardial infarction.

The studies assessed the following NOACs.

- Apixaban
  - 2.5 mg twice daily (BD) and 10 mg once daily (QD) in APPRAISE 1
  - 5 mg BD in APPRAISE 2
- Rivaroxaban
  - 5 mg QD to 20 mg QD in ATLAS ACS
  - 2.5 mg BD and 5 mg BD in ATLAS ACS 2
  - 2.5 mg BD in GEMINI‐ACS
- Dabigatran:
  - 50 mg BD, 75 mg BD, 110 mg BD, and 150 mg BD in REDEEM

All trials evaluated NOACs plus antiplatelet therapy versus placebo plus antiplatelet therapy. In all trials, participants in the NOAC and placebo arms received the same concomitant antiplatelet therapy; however, the antiplatelet regimens differed between trials.

All trials reported all‐cause mortality, cardiovascular mortality, major bleeding, myocardial infarction, and stroke. Three trials provided rates of stent thrombosis (APPRAISE 2, ATLAS ACS 2, GEMINI‐ACS). All trials except REDEEM reported TIMI minor bleeding. Only ATLAS ACS reported systemic embolism. No trials assessed recurrent hospitalisation or health‐related quality of life.

#### Ongoing trials

We identified one ongoing trial, which is a randomised, open‐label, single‐centre trial comparing the effect of three antithrombotic regimens on endogenous fibrinolysis in 150 people with ACS (NCT03775746). People with impaired fibrinolytic status will be randomised to one of three treatment arms: clopidogrel 75 mg QD (Group 1), clopidogrel 75 mg QD plus rivaroxaban 2.5 mg BD (Group 2), and ticagrelor 90 mg BD (Group 3). All participants will also receive aspirin 75 mg QD. Participants will receive rivaroxaban for 30 days. The trialists will assess fibrinolytic status during admission and at two, four, and eight weeks. See the Characteristics of ongoing studies table.

#### Excluded studies

We excluded 19 studies after full‐text assessment: eight had ineligible indications, five had ineligible populations, three had ineligible settings, two had ineligible study design, and one had an ineligible comparator. See the Characteristics of excluded studies table.

### Risk of bias in included studies

Figure 5 and Figure 6 summarise the risk of bias of the included studies. See also the Characteristics of included studies table for further details.

**5:**
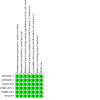
Risk of bias summary: review authors' judgements about each risk of bias item for each included study.

**6:**
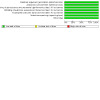
Risk of bias graph: review authors' judgements about each risk of bias item presented as percentages across all included studies.

#### Allocation

All trials randomised participants via an interactive voice‐response system (low risk of bias).

#### Blinding

All trials blinded investigators and participants to treatment assignment in all included trials (low risk of bias).

#### Incomplete outcome data

Participants were analysed in the groups they were randomised to, and losses to follow‐up were low (low risk of bias).

#### Selective reporting

All trials had preregistered protocols and reported all outcomes specified in their protocols (low risk of bias).

#### Other potential sources of bias

There was no indication of other potential sources of bias for any trial (low risk of bias).

### Effects of interventions

See: Table 1; Table 2; Table 3

#### Primary outcomes – primary analyses (NOACs, all doses combined)

For our primary outcomes in the primary analyses involving NOACs of combined doses, there were no closed loops in the network and thus the NMA effect estimates of each 'NOAC (all doses combined) versus placebo' comparison presented below were identical to those of the pairwise meta‐analyses. We did not assess consistency owing to the absence of closed loops in all the networks of our predefined primary outcomes (Primary outcomes).

##### All‐cause mortality

###### Network meta‐analysis

Apixaban (all doses combined) compared with placebo probably has little or no effect on all‐cause mortality (RR 1.09, 95% CI 0.88 to 1.35; NNTH 334; I² = 0%, Tau² = 0; 2 studies, 8638 participants; moderate‐certainty evidence; Table 4). See Analysis 1.1 for the pairwise meta‐analysis effect estimates.

Rivaroxaban (all doses combined) compared with placebo reduces the rate of all‐cause mortality (RR 0.82, 95% CI 0.69 to 0.98; NNTB 250; I² = 0%, Tau² = 0; 3 studies, 21,870 participants; high‐certainty evidence; Table 4). See Analysis 1.2 for the pairwise meta‐analysis effect estimates.

Dabigatran (all doses combined) may reduce the rate of all‐cause mortality compared with placebo (RR 0.57, 95% CI 0.31 to 1.06; NNTB 63; 1 study, 1861 participants; low‐certainty evidence; Table 4). See Analysis 1.3 for the pairwise meta‐analysis effect estimates.

For the outcome all‐cause mortality, apixaban may be inferior to rivaroxaban (RR 1.33, 95% CI 1.01 to 1.76; 5 studies; low‐certainty evidence) and dabigatran (RR 1.92, 95% CI 1.00 to 3.70; 3 studies; low‐certainty evidence). There may be little or no difference in the rate of all‐cause mortality between rivaroxaban and dabigatran (RR 1.45, 95% CI 0.76 to 2.75; 4 studies; low‐certainty evidence). See Figure 1 and Table 4.

##### Cardiovascular mortality

###### Network meta‐analysis

Apixaban (all doses combined) compared with placebo probably has little or no effect on cardiovascular mortality (RR 0.99, 95% CI 0.77 to 1.27; NNT not applicable; I² = 0%, Tau² = 0; 2 studies, 8638 participants; moderate‐certainty evidence; Table 5). See Analysis 2.1 for the pairwise meta‐analysis effect estimates.

Rivaroxaban (all doses combined) compared with placebo probably reduces the rate of cardiovascular mortality (RR 0.83, 95% CI 0.69 to 1.01; NNTB 250; I² = 0%, Tau² = 0; 3 studies, 21,870 participants; moderate‐certainty evidence; Table 5). See Analysis 2.2 for the pairwise meta‐analysis effect estimates.

Dabigatran (all doses combined) compared with placebo may have little or no effect on cardiovascular mortality, although the point estimate suggests a benefit (RR 0.72, 95% CI 0.34 to 1.52; NNTB 143; 1 study, 1861 participants; low‐certainty evidence; Table 5). See Analysis 2.3 for the pairwise meta‐analysis effect estimates.

Low‐certainty evidence suggests little or no difference in the rate of cardiovascular mortality between apixaban and rivaroxaban (RR 1.19, 95% CI 0.87 to 1.62; 5 studies), between apixaban and dabigatran (RR 1.38, 95% CI 0.63 to 3.03; 3 studies), and between rivaroxaban and dabigatran (RR 1.16, 95% CI 0.54 to 2.51; 4 studies). See Figure 1 and Table 5.

##### Major bleeding

###### Network meta‐analysis

Apixaban (all doses combined) increases the rate of major bleeding compared with placebo (RR 2.41, 95% CI 1.44 to 4.06; NNTH 143; I² = 0%, Tau² = 0; 2 studies, 8544 participants; high‐certainty evidence; Table 6). See Analysis 3.1 for the pairwise meta‐analysis effect estimates.

Rivaroxaban (all doses combined) increases the rate of major bleeding compared with placebo (RR 3.31, 95% CI 1.12 to 9.77; NNTH 125; I² = 73%, Tau² = 0.61; 3 studies, 21,870 participants; high‐certainty evidence; Table 6). See Analysis 3.2 for the pairwise meta‐analysis effect estimates.

There may be little or no difference between dabigatran and placebo in the risk of major bleeding (RR 1.74, 95% CI 0.22 to 14.12; NNTH 500; 1 study, 1861 participants; low‐certainty evidence; Table 6). See Analysis 3.3 for the pairwise meta‐analysis effect estimates.

Low‐certainty evidence suggests little or no difference in the rate of major bleeding between apixaban and rivaroxaban (RR 0.67, 95% CI 0.15 to 2.94, 5 studies), between apixaban and dabigatran (RR 1.24, 95% CI 0.08 to 18.21, 3 studies), and between rivaroxaban and dabigatran (RR 1.84, 95% CI 0.14 to 24.75, 4 studies). See Figure 2 and Table 6.

#### Primary outcomes – secondary analyses (different doses of NOACs)

See Appendix 2.

#### Secondary outcomes ‐ secondary analyses (different doses of NOACs)

The results for the secondary outcomes are based on pairwise meta‐analyses.

##### Myocardial infarction

###### NOACs versus placebo

The following investigated doses of apixaban probably have little or no effect on the rate of myocardial infarction compared with placebo (moderate‐certainty evidence; Analysis 4.4).

- All doses combined (RR 0.88, 95% CI 0.67 to 1.16; I² = 17%; 2 studies, 8638 participants)
- 10 mg (RR 0.90, 95% CI 0.71 to 1.14; I² = 5%; 2 studies, 8321 participants)

Apixaban 5 mg may have little or no effect on the rate of myocardial infarction compared with placebo (RR 0.67, 95% CI 0.29 to 1.58; 1 study, 928 participants; low‐certainty evidence; Analysis 4.4).

Rivaroxaban 10 mg reduces the rate of myocardial infarction compared with placebo (RR 0.77, 95% CI 0.65 to 0.92; I² = 0%; 2 studies, 12,444 participants; high‐certainty evidence; Analysis 5.4). The following investigated doses of rivaroxaban probably have little or no effect on the rate of myocardial infarction compared with placebo (moderate‐certainty evidence; Analysis 5.4).

- All doses combined (RR 0.88, 95% CI 0.75 to 1.03; I² = 15%; 3 studies, 21,870 participants)
- 5 mg (RR 0.95, 95% CI 0.81 to 1.11; I² = 0%; 3 studies, 14,732 participants)

The following investigated doses of rivaroxaban may have little or no effect on the rate of myocardial infarction compared with placebo (low‐certainty evidence; Analysis 5.4).

- 15 mg (RR 1.11, 95% CI 0.64 to 1.93; 1 study, 1516 participants)
- 20 mg (RR 0.69, 95% CI 0.40 to 1.19; 1 study, 1771 participants)

The following investigated doses of dabigatran may have little or no effect on the rate of myocardial infarction compared with placebo (low‐certainty evidence; Analysis 6.4).

- All doses combined (RR 1.99, 95% CI 0.71 to 5.60; 1 study, 1861 participants)
- 50 mg BD (RR 2.26, 95% CI 0.70 to 7.28; 1 study, 740 participants)
- 75 mg BD (RR 2.02, 95% CI 0.61 to 6.64; 1 study, 739 participants)
- 110 mg BD (RR 1.60, 95% CI 0.47 to 5.42; 1 study, 777 participants)
- 150 mg BD (RR 2.14, 95% CI 0.65 to 7.04; 1 study, 718 participants)

###### Different doses of NOACs

There may be little or no difference in the rate of myocardial infarction between apixaban 5 mg and apixaban 10 mg (RR 1.17, 95% CI 0.40 to 3.44; 1 study, 635 participants; low‐certainty evidence; Analysis 7.4).

There is probably little or no difference in the rate of myocardial infarction between rivaroxaban 5 mg and rivaroxaban 10 mg (RR 1.17, 95% CI 0.97 to 1.41; I² = 0%; 2 studies, 11,593 participants; moderate‐certainty evidence; Analysis 8.4).

There may be little or no difference in the rate of myocardial infarction between the following doses of rivaroxaban (low‐certainty evidence).

- 5 mg versus 15 mg (RR 0.94, 95% CI 0.46 to 1.92; 1 study, 664 participants; Analysis 9.4)
- 5 mg versus 20 mg (RR 1.52, 95% CI 0.75 to 3.08; 1 study, 919 participants; Analysis 10.4)
- 10 mg versus 15 mg (RR 0.65, 95% CI 0.36 to 1.18; 1 study, 1412 participants; Analysis 11.4)
- 10 mg versus 20 mg (RR 1.06, 95% CI 0.59 to 1.89; 1 study, 1667 participants; Analysis 12.4)
- 15 mg versus 20 mg (RR 1.62, 95% CI 0.83 to 3.16; 1 study, 967 participants; Analysis 13.4)

There may be little or no difference in the rate of myocardial infarction between the following doses of dabigatran (low‐certainty evidence).

- 50 mg BD versus 75 mg BD (RR 1.12, 95% CI 0.44 to 2.88; 1 study, 737 participants; Analysis 14.4)
- 50 mg BD versus 110 mg BD (RR 1.41, 95% CI 0.53 to 3.76; 1 study, 775 participants; Analysis 15.4)
- 50 mg BD versus 150 mg BD (RR 1.06, 95% CI 0.41 to 2.71; 1 study, 716 participants; Analysis 16.4)
- 75 mg BD versus 110 mg BD (RR 1.26, 95% CI 0.46 to 3.44; 1 study, 774 participants; Analysis 17.4)
- 75 mg BD versus 150 mg BD (RR 0.94, 95% CI 0.36 to 2.48; 1 study, 715 participants; Analysis 18.4)
- 110 mg BD versus 150 mg BD (RR 0.75, 95% CI 0.27 to 2.04; 1 study, 753 participants; Analysis 19.4)

##### Stroke

###### NOACs versus placebo

The following investigated doses of apixaban probably have little or no effect on the rate of stroke compared with placebo (moderate‐certainty evidence; Analysis 4.5).

- All doses combined (RR 0.66, 95% CI 0.40 to 1.11; I² = 0%; 2 studies, 8638 participants)
- 10 mg (RR 0.68, 95% CI 0.41 to 1.15; I² = 0%; 2 studies, 8321 participants)

Apixaban 5 mg may have little or no effect on the rate of stroke compared with placebo (RR 0.38, 95% CI 0.02 to 7.99; 1 study, 928 participants; low‐certainty evidence; Analysis 4.5).

The following investigated doses of rivaroxaban probably have little or no effect on the rate of stroke compared with placebo (moderate‐certainty evidence; Analysis 5.5).

- All doses combined (RR 0.84, 95% CI 0.45 to 1.55; I² = 48%; 3 studies, 21,870 participants)
- 5 mg (RR 0.99, 95% CI 0.68 to 1.44; I² = 0%; 3 studies, 14,732 participants)
- 10 mg (RR 1.25, 95% CI 0.85 to 1.83; I² = 0%; 2 studies, 12,444 participants)

The following investigated doses of rivaroxaban may have little or no effect on the rate of stroke compared with placebo (low‐certainty evidence; Analysis 5.5).

- 15 mg (RR 0.25, 95% CI 0.01 to 4.43; 1 study, 1516 participants)
- 20 mg (RR 0.32, 95% CI 0.04 to 2.62; 1 study, 1771 participants)

Dabigatran (all doses combined) may reduce the rate of stroke compared with placebo (RR 0.08, 95% CI 0.01 to 0.80; 1 study, 1861 participants; low‐certainty evidence; Analysis 6.5). The following investigated doses of dabigatran may have little or no effect on the rate of stroke compared with placebo (low‐certainty evidence; Analysis 6.5).

- 50 mg BD (RR 0.14, 95% CI 0.01 to 2.77; 1 study, 740 participants)
- 75 mg BD (RR 0.34, 95% CI 0.04 to 3.22; 1 study, 739 participants)
- 110 mg (RR 0.13, 95% CI 0.01 to 2.52; 1 study, 777 participants)
- 150 mg BD (RR 0.15, 95% CI 0.01 to 2.95; 1 study, 718 participants)

###### Different doses of NOACs

There may be little or no difference in the rate of stroke between apixaban 5 mg and apixaban 10 mg (RR 0.33, 95% CI 0.01 to 8.18; 1 study, 635 participants; low‐certainty evidence; Analysis 7.5).

There is probably little or no difference in the rate of stroke between rivaroxaban 5 mg and rivaroxaban 10 mg (RR 0.85, 95% CI 0.58 to 1.25; I² = 0%; 2 studies, 11,593 participants; moderate‐certainty evidence; Analysis 8.5).

There may be little or no difference in the rate of stroke between the following doses of rivaroxaban (low‐certainty evidence).

- 5 mg versus 15 mg (RR 3.47, 95% CI 0.14 to 84.77; 1 study, 664 participants; Analysis 9.5)
- 5 mg versus 20 mg (RR 1.98, 95% CI 0.12 to 31.61; 1 study, 919 participants; Analysis 10.5)
- 10 mg versus 15 mg (RR 3.04, 95% CI 0.16 to 56.32; 1 study, 1412 participants; Analysis 11.5)
- Rivaroxaban 10 mg versus 20 mg (RR 2.31, 95% CI 0.26 to 20.66; 1 study, 1667 participants; Analysis 12.5)
- Rivaroxaban 15 mg versus 20 mg (RR 0.57, 95% CI 0.02 to 13.99; 1 study, 967 participants; Analysis 13.5)

There may be little or no difference in the rate of stroke between the following doses of dabigatran (low‐certainty evidence).

- 50 mg BD versus 75 mg BD (RR 0.33, 95% CI 0.01 to 8.13; 1 study, 737 participants; Analysis 14.5)
- 75 mg BD versus 110 mg BD (RR 3.31, 95% CI 0.14 to 80.97; 1 study, 774 participants; Analysis 17.5)
- 75 mg versus 150 mg BD (RR 2.83, 95% CI 0.12 to 69.22; 1 study, 715 participants; Analysis 18.5)

##### Stent thrombosis

###### NOACs versus placebo

One RCT compared stent thrombosis between apixaban and placebo (APPRAISE 2). Apixaban 10 mg compared with placebo probably has little or no effect on the rate of stent thrombosis (RR 0.73, 95% CI 0.47 to 1.12; 1 study, 7392 participants; moderate‐certainty evidence; Analysis 4.6).

Two RCTs compared stent thrombosis between rivaroxaban and placebo (ATLAS ACS 2, GEMINI‐ACS). The following investigated doses of rivaroxaban probably have little or no effect on the rate of stent thrombosis compared with placebo (moderate‐certainty evidence; Analysis 5.6).

- All doses combined (RR 0.76, 95% CI 0.52 to 1.12; I² = 27%; 1 study, 18,379 participants)
- 5 mg (RR 0.76, 95% CI 0.49 to 1.19; I² = 35%; 2 studies, 13,264 participants)
- 10 mg (RR 0.71, 95% CI 0.50 to 1.01; 1 study, 10,228 participants)

###### Different doses of NOACs

There is probably little or no difference in the rate of stent thrombosis between rivaroxaban 5 mg and rivaroxaban 10 mg (RR 0.92, 95% CI 0.62 to 1.37; 1 study, 10,229 participants; moderate‐certainty evidence; Analysis 8.6).

##### Non‐major bleeding

###### NOACs versus placebo

The following investigated doses of apixaban increase the rate of non‐major bleeding compared with placebo (high‐certainty evidence; Analysis 4.7).

- All doses combined (RR 2.71, 95% CI 1.47 to 5.01; I² = 0%; 2 studies, 8544 participants)
- 10 mg (RR 2.74, 95% CI 1.45 to 5.17; I² = 0%; 2 studies, 8229 participants)

Apixaban 5 mg may have little or no effect on the rate of non‐major bleeding compared with placebo (RR 1.90, 95% CI 0.39 to 9.37; 1 study, 914 participants; low‐certainty evidence; Analysis 4.7).

The following investigated doses of rivaroxaban increase the rate of non‐major bleeding compared with placebo (high‐certainty evidence; Analysis 5.7).

- All doses combined (RR 2.18, 95% CI 1.41 to 3.35; I² = 0%; 3 studies, 21,870 participants
- 5 mg (RR 1.71, 95% CI 1.04 to 2.80; I² = 0%; 3 studies, 14,732 participants)
- 10 mg (RR 2.52, 95% CI 1.54 to 4.13; I² = 0%; 2 studies, 12,444 participants)

The following investigated doses of rivaroxaban probably increase the rate of non‐major bleeding compared with placebo (moderate‐certainty evidence; Analysis 5.7).

- 15 mg (RR 6.52, 95% CI 1.20 to 35.43; 1 study, 1516 participants)
- 20 mg (RR 4.75, 95% CI 0.92 to 24.39; 1 study, 1771 participants)

###### Different doses of NOACs

There may be little or no difference in the rate of non‐major bleeding between apixaban 5 mg and apixaban 10 mg (RR 1.50, 95% CI 0.25 to 8.92; 1 study, 630 participants; low‐certainty evidence; Analysis 7.6).

There is probably little or no difference in the rate of non‐major bleeding between rivaroxaban 5 mg and rivaroxaban 10 mg (RR 0.65, 95% CI 0.42 to 1.00; I² = 0%; 2 studies, 11,593 participants; moderate‐certainty evidence; Analysis 8.7).

There may be little or no difference in the rate of non‐major bleeding between the following doses of rivaroxaban (low‐certainty evidence).

- 5 mg versus 15 mg (RR 0.29, 95% CI 0.03 to 2.57; 1 study, 664 participants; Analysis 9.6)
- 5 mg versus 20 mg (RR 0.40, 95% CI 0.05 to 3.38; 1 study, 919 participants; Analysis 10.6)
- 10 mg versus 15 mg (RR 0.51, 95% CI 0.14 to 1.78; 1 study, 1412 participants; Analysis 11.6)
- 10 mg versus 20 mg (RR 0.69, 95% CI 0.21 to 2.27; 1 study, 1667 participants; Analysis 12.6)
- 15 mg versus 20 mg (RR 1.37, 95% CI 0.37 to 5.08; 1 study, 967 participants; Analysis 13.6)

##### Recurrent hospitalisation

No studies reported recurrent hospitalisation.

##### Systemic embolism

One RCT assessed systemic embolism between rivaroxaban (5 mg to 20 mg QD) versus placebo (ATLAS ACS). The following investigated doses of rivaroxaban probably have little or no effect on the rate of systemic embolism compared with placebo (moderate‐certainty evidence; Analysis 5.8).

- All doses combined (RR 0.07, 95% CI 0.00 to 1.38; 1 study, 3491 participants)
- 10 mg (RR 0.16, 95% CI 0.01 to 3.03; 1 study, 2216 participants)

The following investigated doses of rivaroxaban may have little or no effect on the rate of systemic embolism compared with placebo (low‐certainty evidence; Analysis 5.8).

- 5 mg (RR 0.54, 95% CI 0.03 to 10.36; 1 study, 1468 participants)
- 15 mg (RR 0.46, 95% CI 0.02 to 8.97; 1 study, 1516 participants)
- 20 mg (RR 0.27, 95% CI 0.01 to 5.24; 1 study, 1771 participants)

##### Health‐related quality of life

No studies reported health‐related quality of life.

#### Subgroup analysis

We found insufficient data to pursue our intended subgroup analyses.

#### Ranking

We ranked competing treatments for the primary outcomes by P scores, which are derived from the P values of all pairwise comparisons, and enable ranking of treatments on a continuous 0‐to‐1 scale. P scores were based solely on the point estimates and standard errors of the frequentist NMA estimates under the normality assumption. P scores measure the mean extent of certainty that a treatment is better than the competing treatments. However, P scores are not a conclusive indicator of treatment performance; they do not reveal the size of treatment effects or the statistical significance of treatment differences. Consequently, it is important to consider other elements when evaluating these outcomes, such as the certainty of evidence and the clinical context.

##### Ranking of treatments (NOACs, all doses combined)

###### All‐cause mortality

The P scores suggest that dabigatran is associated with the lowest risk of all‐cause mortality, followed by rivaroxaban, placebo, and apixaban (Table 8).

**5:** Ranking of treatments according to P values of all pairwise comparisons (non‐vitamin‐K‐antagonist oral anticoagulants, all doses combined versus placebo)

**Intervention**	**Rank (P value)**		
	**All‐cause mortality**	**Cardiovascular death**	**Major bleeding**
**Placebo**	3 (0.2833)	4 (0.2318)	1 (0.8572)
**Apixaban**	4 (0.0838)	3 (0.2943)	3 (0.4108)
**Rivaroxaban**	2 (0.6971)	2 (0.7276)	4 (0.2101)
**Dabigatran**	1 (0.9358)	1 (0.7462)	2 (0.5219)

###### Cardiovascular mortality

The P scores suggest that dabigatran is associated with the lowest risk of cardiovascular mortality, followed by rivaroxaban, apixaban, and placebo (Table 8).

###### Major bleeding

The P scores suggest that placebo is associated with the lowest risk of major bleeding, followed by dabigatran, apixaban, and rivaroxaban (Table 8).

## Discussion

### Summary of main results

Our review aimed to assess the efficacy and safety of NOACs after AMI in people without an indication for anticoagulation. We included six trials, with 33,039 participants, comparing NOACs plus antiplatelet therapy with placebo plus antiplatelet therapy after AMI. To assess the efficacy of these agents, we evaluated all‐cause mortality, cardiovascular mortality, myocardial infarction, stroke, stent thrombosis, recurrent hospitalisation, systemic embolism, and health‐related quality of life. To assess the safety of NOACs, we assessed major TIMI bleeding and any non‐major TIMI bleeding.

#### Efficacy

High‐certainty evidence suggests that rivaroxaban (combined dose) reduces the risk of all‐cause mortality, and moderate‐certainty evidence suggests that rivaroxaban probably reduces the risk of cardiovascular mortality after AMI. Low‐certainty evidence suggests that dabigatran may reduce the rate of all‐cause mortality compared with placebo. Moderate‐certainty evidence suggests no meaningful difference in the rate of all‐cause mortality and cardiovascular mortality between apixaban and placebo. There is uncertainty about the rate of cardiovascular mortality with dabigatran compared with placebo.

There are inconclusive results regarding the efficacy of different doses of NOACs (specifically apixaban, rivaroxaban, and dabigatran) versus placebo for the rate of all‐cause mortality, cardiovascular mortality, stroke, and stent thrombosis. Dabigatran (combined dose) may reduce the risk of stroke compared with placebo. Rivaroxaban (10 mg daily) may reduce the rate of myocardial infarction compared with placebo. Only one trial reported the outcome systemic embolism (ATLAS ACS). No trials assessed recurrent hospitalisation or health‐related quality of life. No trials assessed edoxaban after AMI in people without an indication for oral anticoagulation.

#### Safety

High‐certainty evidence suggests that apixaban and rivaroxaban increase the risk of major bleeding compared with placebo, while moderate‐certainty evidence suggests these drugs probably increase the risk of non‐major bleeding. The evidence is very uncertain about the risk of major bleeding with dabigatran compared with placebo after AMI.

#### Indirect comparisons of different NOACs

We found no head‐to‐head trials of different NOACs. Our NMA compared NOACs agents indirectly against each other, finding that no NOAC was superior to any other at any individual investigated dose for any of the primary outcomes. However, moderate‐certainty evidence suggests that apixaban (combined dose) is probably less effective than rivaroxaban or dabigatran in preventing all‐cause mortality after AMI in people without an indication for anticoagulation.

### Overall completeness and applicability of evidence

We aimed to evaluate the efficacy and safety of NOACs after AMI in people without an indication for anticoagulation. Given the complexity of the condition, and in the absence of RCTs comparing different types of NOACs against each other, we conducted an NMA. This provided a comprehensive, coherent, and methodologically robust comparison of all available treatment options across efficacy and safety outcomes. We combined both direct and indirect evidence, thus increasing the statistical power and confidence in the results.

The conclusions of this review are based on a limited number of RCTs. The included studies reported all of our primary outcomes (all‐cause mortality, cardiovascular mortality, and major bleeding), but not all of our secondary outcomes. Three trials provided rates of stent thrombosis (APPRAISE 2, ATLAS ACS 2, GEMINI‐ACS). All trials except REDEEM reported TIMI minor bleeding. No trials assessed recurrent hospitalisation or health‐related quality of life. Only ATLAS ACS reported systemic embolism. No trials assessed the role of edoxaban in secondary prevention after AMI in people without an indication for anticoagulation.

### Quality of the evidence

The overall certainty of the evidence ranged from low to high. The main reason for downgrading the certainty of the evidence was imprecision of results with wide CIs. Two trials did not meet the optimal information size (APPRAISE 1 and REDEEM).

### Potential biases in the review process

We conducted a comprehensive search for studies and used rigorous methods to minimise bias in the review process. Two review authors independently screened the results of the literature search to identify relevant studies, assessed each included study, extracted data, and assessed the risk of bias using the Cochrane risk of bias tool RoB 1. Any discrepancies between the two review authors were resolved through discussion, and a third reviewer was consulted if necessary.

One strength of our review is that we not only included all published phase II and III RCTs of NOACs, but also retrieved unpublished data related to all individual doses for the phase II study of rivaroxaban. We conducted the review according to a previously published protocol as far as possible; we documented all deviations from the protocol in the Differences between protocol and review section.

However, we acknowledge that our review has some limitations. First, we assessed the outcomes at the latest point of follow‐up for each trial, which ranged from six to 13 months. We identified heterogeneity across the included trials with respect to type of concomitant antiplatelet therapy; follow‐up time; and type, dose, and duration of antithrombotic therapy. This heterogeneity could affect the interpretation of our results. We also acknowledge that most of the participants included in our analysis were part of rivaroxaban trials, and there is less evidence on apixaban and dabigatran. Additionally, the lack of data for the small proportion of people who receive SAPT is a limitation of our review.

Finally, we note that individual participant data were not publicly available. An individual participant‐level data analysis could help us to determine which people would benefit most from a given treatment combination.

Despite these limitations, our review provides valuable insights into the efficacy and safety of NOACs in combination with antiplatelet therapy for secondary prevention after AMI.

### Agreements and disagreements with other studies or reviews

Our findings agree with and extend the findings of three previous systematic reviews.

Oldgren 2013 performed a meta‐analysis of RCTs to evaluate the efficacy and safety of adding NOACs (apixaban, dabigatran, darexaban, rivaroxaban, and ximelagatran) to single (aspirin) or dual (aspirin and clopidogrel) antiplatelet therapy after ACS. The findings suggested that adding NOACs to antiplatelet therapy resulted in a modest reduction in cardiovascular events but a substantial increase in bleeding. However, Oldgren 2013 included RCTs of NOACs that were not approved by the FDA (ximelagatran and darexaban).

Khan 2017 conducted a meta‐analysis to assess the safety and efficacy of adding NOACs (apixaban, rivaroxaban, and dabigatran) to SAPT or DAPT in people with ACS, and concluded that the addition of NOACs to DAPT was associated with an increase in the risk of clinically significant bleeding and only a modest reduction in major adverse cardiovascular events. The addition of NOACs to SAPT did not result in a significant reduction in major adverse cardiovascular events or an increase in clinically significant bleeding. However, Khan 2017 included RCTs assessing NOACs in people with an indication for anticoagulation due to atrial fibrillation.

Chiarito 2018 suggested a favourable net clinical benefit when adding NOACs to antiplatelet therapy for secondary prevention after ACS, particularly in people presenting with STEMI; the findings showed that administration of NOACs in addition to antiplatelet therapy after STEMI appeared to improve ischaemic events at the cost of a marginally increased risk of major bleeding.

In contrast to these previous meta‐analyses, we used individual efficacy outcomes rather than composite outcomes, which might explain why we found no meaningful difference in efficacy for all individual doses. We analysed the safety results using the TIMI criteria to avoid the limitation of the variability in definitions of bleeding events across included studies. Furthermore, our review provided a comprehensive and comparative evaluation of all available treatment options within an NMA framework, thus increasing the statistical power and confidence in the results.

## Authors' conclusions

**Implications for practice**

Compared with placebo, non‐vitamin K antagonist oral anticoagulants (NOACs; specifically apixaban and rivaroxaban) in addition to antiplatelet therapy after acute myocardial infarction (AMI) in people without an indication for oral anticoagulation are associated with increased risk of major bleeding. Rivaroxaban compared to placebo reduces the risk of all‐cause mortality and probably reduces the risk of cardiovascular mortality. However, we detected no meaningful difference in efficacy outcomes for any of the NOACs at specific doses compared to placebo.

**Implications for research**

Although the evidence suggests that NOACs reduce mortality, the effect size/impact is small and associated with increased bleeding. Our data show that clinicians should exercise caution when considering NOACs as a therapeutic option for people who have had an AMI, particularly in view of the widespread use of potent P2Y12 inhibitors. More research is required to better understand the appropriate use of NOACs in this population. The available evidence does not support the hypothesis that higher NOAC doses result in a greater reduction of ischaemic events. This finding could affect future trial design and dosage selection. Lower NOAC doses paired with a single antiplatelet therapy might be a safe strategy after AMI. However, more research is needed to determine the benefits of this regimen in terms of efficacy outcomes compared with antiplatelet therapy alone. In addition, future studies should aim to determine which people would benefit from the addition of a NOAC to antiplatelets. The results of this review suggest that an appropriate target population may be people with higher atherothrombotic risk who are not at increased risk for bleeding. Identifying this subpopulation represents a challenge for future research.

Outcomes of future studies should include risk of recurrent hospitalisation and health‐related quality of life. Almost all included trials were conducted while clopidogrel was the sole P2Y12 inhibitor available. Therefore, researchers should compare NOACs with potent P2Y12 inhibitors (prasugrel and ticagrelor) to establish a regimen with an improved efficacy/safety profile for people at high ischaemic risk. In addition, there is a need for head‐to‐head trials of different NOACs to determine the preferred NOAC agent in antithrombotic therapy that combines platelet inhibition and anticoagulation after AMI.

## History

Protocol first published: Issue 5, 2021

## Appendices

### Appendix 1. Search strategies

#### CENTRAL

#1 MeSH descriptor: [Myocardial Infarction] explode all trees

#2 Myocardial infarction

#3 (MI or AMI)

#4 ST‐segment elevation myocardial infarction

#5 non‐ST segment elevation myocardial infarction

#6 (NSTEMI or STEMI)

#7 heart attack*

#8 {OR #1‐#7}

#9 ((novel or new) NEAR/5 anticoagulant*)

#10 ((non‐vitamin K or direct) NEAR/5 oral anticoagulant*)

#11 NOACS

#12 DOACS

#13 Apixaban

#14 MeSH descriptor: [Dabigatran] this term only

#15 Dabigatran

#16 Edoxaban

#17 MeSH descriptor: [Rivaroxaban] this term only

#18 Rivaroxaban

#19 {OR #9‐#18}

#20 #8 AND #19

#### MEDLINE Ovid

1 exp Myocardial Infarction/ (180783)

2 Myocardial infarction.tw. (187199)

3 (MI or AMI).tw. (69445)

4 ST‐segment elevation myocardial infarction.tw. (9027)

5 non‐ST segment elevation myocardial infarction.tw. (1688)

6 (NSTEMI or STEMI).tw. (13276)

7 heart attack*.tw. (5811)

8 1 or 2 or 3 or 4 or 5 or 6 or 7 (284530)

9 ((novel or new) adj5 anticoagulant*).tw. (5210)

10 ((non‐vitamin K or direct) adj5 oral anticoagulant*).tw. (5682)

11 NOACS.tw. (1852)

12 DOACS.tw. (2124)

13 Apixaban.tw. (3629)

14 Dabigatran/ (3365)

15 dabigatran.tw. (4991)

16 Edoxaban.tw. (1516)

17 Rivaroxaban/ (3720)

18 rivaroxaban.tw. (5636)

19 9 or 10 or 11 or 12 or 13 or 14 or 15 or 16 or 17 or 18 (17181)

20 8 and 19 (947)

21 randomized controlled trial.pt. (534678)

22 controlled clinical trial.pt. (94229)

23 randomized.ab. (524225)

24 placebo.ab. (219053)

25 drug therapy.fs. (2336544)

26 randomly.ab. (360014)

27 trial.ab. (556623)

28 groups.ab. (2209947)

29 21 or 22 or 23 or 24 or 25 or 26 or 27 or 28 (5037851)

30 exp animals/ not humans.sh. (4849891)

31 29 not 30 (4380044)

32 20 and 31 (718)

#### Embase Ovid

1 exp heart infarction/ (381377)

2 Myocardial infarction.tw. (267719)

3 (MI or AMI).tw. (121925)

4 ST‐segment elevation myocardial infarction.tw. (14379)

5 non‐ST segment elevation myocardial infarction.tw. (2547)

6 (NSTEMI or STEMI).tw. (33786)

7 heart attack*.tw. (8223)

8 1 or 2 or 3 or 4 or 5 or 6 or 7 (463386)

9 ((novel or new) adj5 anticoagulant*).tw. (8835)

10 ((non‐vitamin K or direct) adj5 oral anticoagulant*).tw. (9600)

11 NOACS.tw. (3855)

12 DOACS.tw. (4130)

13 apixaban/ (14352)

14 Apixaban.tw. (7833)

15 dabigatran/ (15298)

16 Dabigatran.tw. (10162)

17 edoxaban/ (5405)

18 Edoxaban.tw. (2744)

19 rivaroxaban/ (20404)

20 Rivaroxaban.tw. (11776)

21 9 or 10 or 11 or 12 or 13 or 14 or 15 or 16 or 17 or 18 or 19 or 20 (39822)

22 8 and 21 (4020)

23 random$.tw. (1662380)

24 factorial$.tw. (40743)

25 crossover$.tw. (79103)

26 cross over$.tw. (33121)

27 cross‐over$.tw. (33121)

28 placebo$.tw. (321543)

29 (doubl$ adj blind$).tw. (214008)

30 (singl$ adj blind$).tw. (26845)

31 assign$.tw. (420769)

32 allocat$.tw. (167033)

33 volunteer$.tw. (262761)

34 crossover procedure/ (67244)

35 double blind procedure/ (182224)

36 randomized controlled trial/ (658353)

37 single blind procedure/ (42924)

38 23 or 24 or 25 or 26 or 27 or 28 or 29 or 30 or 31 or 32 or 33 or 34 or 35 or 36 or 37 (2479777)

39 (animal/ or nonhuman/) not human/ (5815400)

40 38 not 39 (2198481)

41 22 and 40 (1035)

#### CPCI‐S

# 16 #15 AND #14

# 15 TS=(random* or blind* or allocat* or assign* or trial* or placebo* or crossover* or cross‐over*)

# 14 #13 AND #7

# 13 #12 OR #11 OR #10 OR #9 OR #8

# 12 TS=(Apixaban or Dabigatran or Edoxaban or Rivaroxaban)

# 11 TS=DOACS

# 10 TS=NOACS

# 9 TS=(("non‐vitamin K" or direct) NEAR/5 "oral anticoagulant*")

# 8 TS=((novel or new) NEAR/5 anticoagulant*)

# 7 #6 OR #5 OR #4 OR #3 OR #2 OR #1

# 6 TS=heart attack*

# 5 TS=(NSTEMI or STEMI)

# 4 TS=non‐ST segment elevation myocardial infarction

# 3 TS=ST‐segment elevation myocardial infarction

# 2 TS=(MI or AMI)

# 1 TS=Myocardial infarction

#### ClinicalTrials.gov

Advanced search

Condition or disease: Acute Myocardial Infarction

AND

Study type: Interventional Studies (Clinical Trials)

AND

Intervention/treatment: Anticoagulant

#### WHO ICTRP

Advanced search

Condition: Acute Myocardial Infarction (with synonyms)

AND

Intervention: Anticoagulants (with synonyms)

### Appendix 2. Primary outcomes – secondary analyses (different doses of NOACs)

#### All‐cause mortality

##### Non‐vitamin‐K‐antagonist oral anticoagulants (NOACs) at different doses versus placebo

###### Direct evidence

There may be little or no difference in the rate of all‐cause mortality between apixaban 5 mg and placebo (RR 1.77, 95% CI 0.79 to 3.96; 1 study, 928 participants; low‐certainty evidence; Analysis 4.1), and there is probably little or no difference in the rate of all‐cause mortality between apixaban 10 mg and placebo (RR 1.06, 95% CI 0.86 to 1.32; I² = 0%; 2 studies, 8321 participants; moderate‐certainty evidence; Analysis 4.1).

The following doses of rivaroxaban probably have little or no effect on the rate of all‐cause mortality compared with placebo (moderate‐certainty evidence; Analysis 5.1).

- 5 mg (RR 1.00, 95% CI 0.55 to 1.82; I² = 74%; 3 studies, 14,732 participants)
- 10 mg (RR 0.84, 95% CI 0.56 to 1.26; I² = 28%; 2 studies, 12,444 participants)

The following doses of rivaroxaban may have little or no effect on the rate of all‐cause mortality compared with placebo (low‐certainty evidence; Analysis 5.1).

- 15 mg (RR 0.61, 95% CI 0.18 to 2.08; I² = 0%; 1 study, 1516 participants)
- 20 mg (RR 1.07, 95% CI 0.47 to 2.40; 1 study, 1771 participants)

The following doses of dabigatran may have little or no effect on the rate of all‐cause mortality compared with placebo (low‐certainty evidence; Analysis 6.1).

- 50 mg BD (RR 0.57, 95% CI 0.24 to 1.35; 1 study, 740 participants)
- 75 mg BD (RR 0.72, 95% CI 0.32 to 1.60; 1 study, 739 participants)
- 110 mg BD (RR 0.46, 95% CI 0.19 to 1.12; 1 study, 777 participants)
- Dabigatran 150 mg BD (RR 0.53, 95% CI 0.22 to 1.31; 1 study, 718 participants)

###### Network meta‐analysis

The following doses of apixaban may have little or no effect on the rate of all‐cause mortality compared with placebo (low‐certainty evidence).

- 5 mg (RR 1.91, 95% CI 0.62 to 5.86)
- 10 mg (RR 0.98, 95% CI 0.46 to 2.10)

The following doses of rivaroxaban may have little or no effect on the rate of all‐cause mortality compared with placebo (low‐certainty evidence).

- 5 mg (RR 0.99, 95% CI 0.54 to 1.82)
- 10 mg (RR 0.79, 95% CI 0.39 to 1.60)
- 15 mg (RR 0.54, 95% CI 0.13 to 2.22)
- 20 mg (RR 0.94, 95% CI 0.32 to 2.77)

The following doses of dabigatran may have little or no effect on the rate of all‐cause mortality compared with placebo (low‐certainty evidence).

- 50 mg BD (RR 0.57, 95% CI 0.17 to 1.98)
- 75 mg BD (RR 0.72, 95% CI 0.22 to 2.38)
- 110 mg BD (RR 0.46, 95% CI 0.13 to 1.62)
- 150 mg BD (RR 0.53, 95% CI 0.15 to 1.89)

##### NOACs at different doses compared to each other

###### Network meta‐analysis

There may be little or no difference in the rate of all‐cause mortality between apixaban 5 mg and apixaban 10 mg (RR 2.21, 95% CI 0.78 to 6.28; 1 study, 635 participants; low‐certainty evidence; Analysis 7.1).

There is probably little or no difference in the rate of all‐cause mortality between rivaroxaban 5 mg and rivaroxaban 10 mg (RR 1.57, 95% CI 0.31 to 8.03; I² = 91%; 2 studies, 11,593 participants; moderate‐certainty evidence; Analysis 8.1). There may be little or no difference in the rate of all‐cause mortality between the following doses of rivaroxaban (low‐certainty evidence).

- 5 mg versus 15 mg (RR 3.47, 95% CI 0.95 to 12.69; 1 study, 664 participants; Analysis 9.1)
- 5 mg versus 20 mg (RR 1.98, 95% CI 0.80 to 4.95; 1 study, 919 participants; Analysis 10.1)
- 10 mg versus 15 mg (RR 0.90, 95% CI 0.24 to 3.37; 1 study, 1412 participants; Analysis 11.1)
- 10 mg versus 20 mg (RR 0.51, 95% CI 0.20 to 1.33; 1 study, 1667 participants; Analysis 12.1)
- 15 mg versus 20 mg (RR 0.57, 95% CI 0.16 to 2.10; 1 study, 967 participants; Analysis 13.1)

There may be little or no difference in the rate of all‐cause mortality between the following doses of dabigatran (low‐certainty evidence)

- 50 mg BD versus 75 mg BD (RR 0.80, 95% CI 0.32 to 2.00; 1 study, 737 participants; Analysis 14.1)
- 50 mg BD versus 110 mg BD (RR 1.26, 95% CI 0.46 to 3.43; 1 study, 775 participants; Analysis 15.1)
- 50 mg BD versus 150 mg BD (RR 1.07, 95% CI 0.39 to 2.93; 1 study, 715 participants; Analysis 16.1)
- 75 mg BD versus 110 mg BD (RR 1.58, 95% CI 0.61 to 4.10; 1 study, 774 participants; Analysis 17.1)
- 75 mg BD versus 150 mg BD (RR 1.35, 95% CI 0.52 to 3.50; 1 study, 715 participants; Analysis 18.1)
- 110 mg BD versus 150 mg BD (RR 0.85, 95% CI 0.30 to 2.41; 1 study, 753 participants; Analysis 19.1).

There may be little or no difference in the rate of all‐cause mortality between apixaban 5 mg and the following interventions (low‐certainty evidence; Figure 3).

- Rivaroxaban 5 mg (RR 1.92, 95% CI 0.54 to 6.86)
- Rivaroxaban 10 mg (RR 2.43, 95% CI 0.65 to 9.14)
- Rivaroxaban 15 mg (RR 3.57, 95% CI 0.58 to 21.83)
- Rivaroxaban 20 mg (RR 2.04, 95% CI 0.43 to 9.71)
- Dabigatran 50 mg BD (RR 3.33, 95% CI 0.63 to 17.62)
- Dabigatran 75 mg BD (RR 2.65, 95% CI 0.52 to 13.66)
- Dabigatran 110 mg BD (RR 4.18, 95% CI 0.77 to 22.62)
- Dabigatran 150 mg BD (RR 3.57, 95% CI 0.66 to 19.33)

There may be little or no difference in the rate of all‐cause mortality between apixaban 10 mg and the following interventions (low‐certainty evidence; Figure 3).

- Rivaroxaban 5 mg (RR 0.99, 95% CI 0.37 to 2.61)
- Rivaroxaban 10 mg (RR 1.25, 95% CI 0.44 to 3.53)
- Rivaroxaban 15 mg (RR 1.84, 95% CI 0.37 to 9.22)
- Rivaroxaban 20 mg (RR 1.05, 95% CI 0.28 to 3.95)
- Dabigatran 50 mg BD (RR 1.71, 95% CI 0.40 to 7.30)
- Dabigatran 75 mg BD (RR 1.37, 95% CI 0.33 to 5.63)
- Dabigatran 110 mg BD (RR 2.15, 95% CI 0.49 to 9.40)
- Dabigatran 150 mg BD (RR 1.84, 95% CI 0.42 to 8.03)

There may be little or no difference in the rate of all‐cause mortality between rivaroxaban 5 mg and the following interventions (low‐certainty evidence; Figure 3).

- Dabigatran 50 mg BD (RR 1.73, 95% CI 0.44 to 6.84)
- Dabigatran 75 mg BD (RR 1.38, 95% CI 0.36 to 5.27)
- Dabigatran 110 mg BD (RR 2.18, 95% CI 0.54 to 8.82)
- Dabigatran 150 mg BD (RR 1.86, 95% CI 0.46 to 7.53)

There may be little or no difference in the rate of all‐cause mortality between rivaroxaban 10 mg and the following interventions (low‐certainty evidence; Figure 3).

- Dabigatran 50 mg BD (RR 1.37, 95% CI 0.33 to 5.68)
- Dabigatran 75 mg BD (RR 1.09, 95% CI 0.27 to 4.38)
- Dabigatran 110 mg BD (RR 1.72, 95% CI 0.41 to 7.32)
- Dabigatran 150 mg BD (RR 1.47, 95% CI 0.35 to 6.25)

There may be little or no difference in the rate of all‐cause mortality between rivaroxaban 15 mg and the following interventions (low‐certainty evidence; Figure 3).

- Dabigatran 50 mg BD (RR 0.93, 95% CI 0.14 to 6.14)
- Dabigatran 75 mg BD (RR 0.74, 95% CI 0.12 to 4.77)
- Dabigatran 110 mg BD (RR 1.17, 95% CI 0.17 to 7.86)
- Dabigatran 150 mg BD (RR 1.00, 95% CI 0.15 to 6.71)

There may be little or no difference in the rate of all‐cause mortality between rivaroxaban 20 mg and the following interventions (low‐certainty evidence; Figure 3).

- Dabigatran 50 mg BD (RR 1.63, 95% CI 0.31 to 8.43)
- Dabigatran 75 mg BD (RR 1.30, 95% CI 0.26 to 6.53)
- Dabigatran 110 mg BD (RR 2.05, 95% CI 0.39 to 10.83)
- Dabigatran 150 mg BD (RR 1.75, 95% CI 0.33 to 9.25)

#### Cardiovascular mortality

##### NOACs at different doses versus placebo

###### Direct evidence

There may be little or no difference in the rate of cardiovascular mortality between apixaban 5 mg and placebo (RR 1.93, 95% CI 0.84 to 4.40; 1 study, 928 participants; low‐certainty evidence; Analysis 4.2). There is probably little or no difference in the rate of cardiovascular mortality between apixaban 10 mg and placebo (RR 0.94, 95% CI 0.73 to 1.22; I² = 0%; 2 studies, 8321 participants; moderate‐certainty evidence; Analysis 4.2).

The following doses of rivaroxaban probably have little or no effect on the rate of cardiovascular mortality compared with placebo (moderate‐certainty evidence; Analysis 5.2).

- 5 mg (RR 1.14, 95% CI 0.53 to 2.44; I² = 81%; 3 studies, 14,732 participants)
- 10 mg (RR 0.90, 95% CI 0.72 to 1.13; I² = 0%; 2 studies, 12,444 participants)

The following doses of rivaroxaban may have little or no effect on the rate of cardiovascular mortality compared with placebo (low‐certainty evidence; Analysis 5.2)

- 15 mg (RR 0.75, 95% CI 0.22 to 2.62; 1 study, 1516 participants)
- 20 mg (RR 1.17, 95% CI 0.49 to 2.80; 1 study, 1771 participants)

The following doses of dabigatran may have little or no effect on the rate of cardiovascular mortality compared with placebo (low‐certainty evidence; Analysis 6.2).

- 50 mg BD (RR 0.89, 95% CI 0.35 to 2.29; 1 study, 740 participants)
- 75 mg BD (RR 1.01, 95% CI 0.40 to 2.51; 1 study, 739 participants)
- 110 mg BD (RR 0.51, 95% CI 0.17 to 1.50; 1 study, 777 participants)
- 150 mg BD (RR 0.48, 95% CI 0.15 to 1.53; 1 study, 718 participants)

###### Network meta‐analysis

The following doses of apixaban may have little or no effect on the rate of cardiovascular mortality compared with placebo (low‐certainty evidence).

- 5 mg (RR 1.98, 95% CI 0.60 to 6.55)
- 10 mg (RR 0.93, 95% CI 0.40 to 2.14)

The following doses of rivaroxaban may have little or no effect on the rate of cardiovascular mortality compared with placebo (low‐certainty evidence).

- 5 mg (RR 1.07, 95% CI 0.55 to 2.07)
- 10 mg (RR 0.81, 95% CI 0.37 to 1.75)
- 15 mg (RR 0.57, 95% CI 0.13 to 2.48)
- 20 mg (RR 0.88, 95% CI 0.27 to 2.85)

The following doses of dabigatran may have little or no effect on the rate of cardiovascular mortality compared with placebo (low‐certainty evidence).

- 50 mg BD (RR 0.89, 95% CI 0.23 to 3.50)
- 75 mg BD (RR 1.01, 95% CI 0.26 to 3.87)
- 110 mg BD (RR 0.51, 95% CI 0.12 to 2.20)
- 150 mg BD (RR 0.48, 95% CI 0.10 to 2.20)

##### NOACs compared to each other

###### Network meta‐analysis

There may be little or no difference in the rate of cardiovascular mortality between apixaban 5 mg and apixaban 10 mg (RR 2.76, 95% CI 0.89 to 8.57; 1 study, 635 participants; low‐certainty evidence; Analysis 7.2).

There is probably little or no difference in the rate of cardiovascular mortality between rivaroxaban 5 mg and rivaroxaban 10 mg (RR 1.56, 95% CI 0.30 to 8.11; I² = 91%; 2 studies, 11,593 participants; moderate‐certainty evidence; Analysis 8.2) There may be little or no difference in the rate of cardiovascular mortality between the following doses of rivaroxaban (low‐certainty evidence).

- 5 mg versus 15 mg (RR 3.47, 95% CI 0.95 to 12.69; 1 study, 664 participants; Analysis 9.2)
- 5 mg versus 20 mg (RR 2.23, 95% CI 0.87 to 5.73; 1 study, 919 participants; low‐certainty evidence; Analysis 10.2)
- 10 mg versus 15 mg (RR 0.90, 95% CI 0.24 to 3.37; 1 study, 1412 participants; low‐certainty evidence; Analysis 11.2)
- 10 mg versus 20 mg (RR 0.58, 95% CI 0.22 to 1.53; 1 study 1667 participants; low‐certainty evidence; Analysis 12.2)
- 15 mg versus 20 mg (RR 0.64, 95% CI 0.17 to 2.41; 1 study, 967 participants; low‐certainty evidence; Analysis 13.2)

There may be little or no difference in the rate of cardiovascular mortality between the following doses of dabigatran (low‐certainty evidence).

- 50 mg BD versus 75 mg BD (RR 0.89, 95% CI 0.35 to 2.27; 1 study, 737 participants; low‐certainty evidence; Analysis 14.2)
- 50 mg BD versus 110 mg BD (RR 1.76, 95% CI 0.58 to 5.33; 1 study, 775 participants; low‐certainty evidence; Analysis 15.2)
- 50 mg BD versus 150 mg BD (RR 1.88, 95% CI 0.57 to 6.19; 1 study, 716 participants; low‐certainty evidence; Analysis 16.2)
- 75 mg BD versus 110 mg BD (RR 1.99, 95% CI 0.67 to 5.87; 1 study, 774 participants; low‐certainty evidence; Analysis 17.2)
- 75 mg BD versus 150 mg BD (RR 2.12, 95% CI 0.66 to 6.83; 1 study, 715 participants; low‐certainty evidence; Analysis 18.2)
- 110 mg BD versus 150 mg BD (RR 1.07, 95% CI 0.29 to 3.95; 1 study, 753 participants; low‐certainty evidence; Analysis 19.2)

There may be little or no difference in the rate of cardiovascular mortality between apixaban 5 mg and the following interventions (low‐certainty evidence; Figure 3).

- Rivaroxaban 5 mg (RR 1.85, 95% CI 0.47 to 7.27)
- Rivaroxaban 10 mg (RR 2.45, 95% CI 0.59 to 10.18)
- Rivaroxaban 15 mg (RR 3.49, 95% CI 0.52 to 23.41)
- Rivaroxaban 20 mg (RR 2.25, 95% CI 0.42 to 12.07)
- Dabigatran 50 mg BD (RR 2.21, 95% CI 0.36 to 13.60)
- Dabigatran 75 mg BD (RR 1.96, 95% CI 0.32 to 11.88)
- Dabigatran 110 mg BD (RR 3.89, 95% CI 0.59 to 25.88)
- Dabigatran 150 mg BD (RR 4.16, 95% CI 0.60 to 29.06)

There may be little or no difference in the rate of cardiovascular mortality between apixaban 10 mg and the following interventions (low‐certainty evidence; Figure 3).

- Rivaroxaban 5 mg (RR 0.87, 95% CI 0.30 to 2.52)
- Rivaroxaban 10 mg (RR 1.15, 95% CI 0.37 to 3.59)
- Rivaroxaban 15 mg (RR 1.64, 95% CI 0.30 to 8.96)
- Rivaroxaban 20 mg (RR 1.06, 95% CI 0.25 to 4.48)
- Dabigatran 50 mg BD (RR 1.04, 95% CI 0.21 to 5.15)
- Dabigatran 75 mg BD (RR 0.92, 95% CI 0.19 to 4.49)
- Dabigatran 110 mg BD (RR 1.83, 95% CI 0.34 to 9.90)
- Dabigatran 150 mg BD (RR 1.96, 95% CI 0.34 to 11.18)

There may be little or no difference in the rate of cardiovascular mortality between rivaroxaban 5 mg and the following interventions (low‐certainty evidence; Figure 3).

- Dabigatran 50 mg BD (RR 1.20, 95% CI 0.26 to 5.46)
- Dabigatran 75 mg BD (RR 1.06, 95% CI 0.24 to 4.75)
- Dabigatran 110 mg BD (RR 2.11, 95% CI 0.42 to 10.53)
- Dabigatran 150 mg BD (RR 2.25, 95% CI 0.42 to 11.93)

There may be little or no difference in the rate of cardiovascular mortality between rivaroxaban 10 mg and the following interventions (low‐certainty evidence; Figure 3).

- Dabigatran 50 mg BD (RR 0.90, 95% CI 0.19 to 4.34)
- Dabigatran 75 mg BD (RR 0.80, 95% CI 0.17 to 3.78)
- Dabigatran 110 mg BD (RR 1.59, 95% CI 0.30 to 8.35)
- Dabigatran 150 mg BD (RR 1.70, 95% CI 0.31 to 9.44)

There may be little or no difference in the rate of cardiovascular mortality between rivaroxaban 15 mg and the following interventions (low‐certainty evidence; Figure 3).

- Dabigatran 50 mg BD (RR 0.63, 95% CI 0.08 to 4.73)
- Dabigatran 75 mg BD (RR 0.56, 95% CI 0.08 to 4.14)
- Dabigatran 110 mg BD (RR 1.11, 95% CI 0.14 to 8.93)
- Dabigatran 150 mg BD (RR 1.19, 95% CI 0.14 to 9.98)

There may be little or no difference in the rate of cardiovascular mortality between rivaroxaban 20 mg and the following interventions (low‐certainty evidence; Figure 3).

- Dabigatran 50 mg BD (RR 0.98, 95% CI 0.16 to 5.96)
- Dabigatran 75 mg BD (RR 0.87, 95% CI 0.15 to 5.21)
- Dabigatran 110 mg BD (RR 1.73, 95% CI 0.26 to 11.35)
- Dabigatran 150 mg BD (RR 1.85, 95% CI 0.27 to 12.75)

#### Major bleeding

##### NOACs at different doses versus placebo

###### Direct evidence

Apixaban 10 mg increases the rate of major bleeding compared with placebo (RR 2.56, 95% CI 1.52 to 4.30; I² = 0%; 2 studies, 8229 participants; high‐certainty evidence; Analysis 4.3). There may be little or no difference between apixaban 5 mg and placebo in risk of major bleeding (RR 0.38, 95% CI 0.02 to 7.89; 1 study, 914 participants; low‐certainty evidence; Analysis 4.3).

Rivaroxaban at the following investigated doses increases the rate of major bleeding compared with placebo (high‐certainty evidence; Analysis 5.3).

- 5 mg (RR 2.39, 95% CI 1.11 to 5.16; I² = 44%; 3 studies, 14,732 participants)
- 10 mg (RR 6.17, 95% CI 1.83 to 20.85; I² = 45%; 2 studies, 12,444 participants)

Rivaroxaban at the following investigated doses probably increases the rate of major bleeding compared with placebo (moderate‐certainty evidence; Analysis 5.3).

- 15 mg (RR 19.55, 95% CI 2.36 to 161.85; 1 study, 1516 participants)
- 20 mg (RR 15.19, 95% CI 1.90 to 121.15; 1 study, 1771 participants)

There may be little or no difference in major bleeding between the following doses of dabigatran and placebo (low‐certainty evidence; Analysis 6.3).

- 50 mg BD (RR 1.01, 95% CI 0.06 to 16.01; 1 study, 740 participants)
- 75 mg BD (RR 0.34, 95% CI 0.01 to 8.22; 1 study, 739 participants)
- 110 mg BD (RR 4.57, 95% CI 0.54 to 38.93; 1 study, 777 participants)
- 150 mg BD (RR 1.07, 95% CI 0.07 to 17.03; 1 study, 718 participants).

###### Network meta‐analysis

Apixaban 10 mg probably increases the rate of major bleeding compared with placebo (RR 2.57, 95% CI 1.00 to 6.56; moderate‐certainty evidence). We are uncertain about the effect of apixaban 5 mg on major bleeding (RR 0.37, 95% CI 0.02 to 7.80; very low‐certainty evidence).

Rivaroxaban at the following investigated doses probably increases the rate of major bleeding compared with placebo (moderate‐certainty evidence).

- 5 mg (RR 2.42, 95% CI 1.09 to 5.38)
- 10 mg (RR 4.77, 95% CI 1.95 to 11.65)
- 15 mg (RR 6.89, 95% CI 1.61 to 29.52
- 20 mg (RR 5.35, 95% CI 1.32 to 21.72)

We are uncertain about the effect of the following investigated doses of dabigatran on major bleeding compared with placebo (very low‐certainty evidence).

- 50 mg BD (RR 1.01, 95% CI 0.09 to 11.61)
- 75 mg BD (RR 0.34, 95% CI 0.01 to 9.41)
- 110 mg BD (RR 3.35, 95% CI 0.44 to 25.53)
- 150 mg BD (RR 1.07, 95% CI 0.09 to 12.34)

##### NOACs at different doses compared to each other

###### Network meta‐analysis

There may be little or no difference in the rate of major bleeding between apixaban 5 mg and apixaban 10 mg (RR 0.14, 95% CI 0.01 to 2.75; 1 study, 630 participants; low‐certainty evidence; Analysis 7.3).

There is probably little or no difference in the rate of major bleeding between rivaroxaban 5 mg and rivaroxaban 10 mg (RR 0.61, 95% CI 0.21 to 1.72; I² = 38%; 2 studies, 11,593 participants; moderate‐certainty evidence; Analysis 8.3). There may be little or no difference in the rate of major bleeding between the following doses of rivaroxaban and placebo (low‐certainty evidence).

- 5 mg versus 15 mg (RR 0.19, 95% CI 0.02 to 1.59; 1 study, 664 participants; Analysis 9.3)
- 5 mg versus 20 mg (RR 0.25, 95% CI 0.03 to 1.97; 1 study, 919 participants; Analysis 10.3)
- 10 mg versus 15 mg (RR 0.90, 95% CI 0.35 to 2.28; 1 study, 1412 participants; Analysis 11.3)
- 10 mg versus 20 mg (RR 1.16, 95% CI 0.50 to 2.69; 1 study, 1667 participants; Analysis 12.3)
- 15 mg versus 20 mg (RR 1.29, 95% CI 0.45 to 3.68; 1 study, 967 participants; Analysis 13.3)

There may be little or no difference in the rate of major bleeding between the following doses of dabigatran (low‐certainty evidence).

- 50 mg BD versus 75 mg BD (RR 2.99, 95% CI 0.12 to 73.21; 1 study, 737 participants; low‐certainty evidence; Analysis 14.3)
- 50 mg BD versus 110 mg BD (RR 0.22, 95% CI 0.03 to 1.87; 1 study, 775 participants; low‐certainty evidence; Analysis 15.3)
- 50 mg BD versus 150 mg BD (RR 0.94, 95% CI 0.06 to 14.98; 1 study, 716 participants; low‐certainty evidence; Analysis 16.3)
- 75 mg BD versus 110 mg BD (RR 0.10, 95% CI 0.01 to 1.81; 1 study, 774 participants; low‐certainty evidence; Analysis 17.3)
- 75 mg BD versus 150 mg BD (RR 0.31, 95% CI 0.01 to 7.69; 1 study, 715 participants; low‐certainty evidence; Analysis 18.3)
- 110 mg BD versus 150 mg BD (RR 4.27, 95% CI 0.50 to 36.40; 1 study, 753 participants; low‐certainty evidence; Analysis 19.3)

There may be little or no difference in the rate of major bleeding between apixaban 5 mg and the following interventions (low‐certainty evidence; Figure 3).

- Rivaroxaban 5 mg (RR 0.15, 95% CI 0.01 to 3.58)
- Rivaroxaban 10 mg (RR 0.08, 95% CI 0.00 to 1.86)
- Rivaroxaban 15 mg (RR 0.05, 95% CI 0.00 to 1.58)
- Rivaroxaban 20 mg (RR 0.07, 95% CI 0.00 to 1.98)
- Dabigatran 50 mg BD (RR 0.37, 95% CI 0.01 to 18.36)
- Dabigatran 75 mg BD (RR 1.11, 95% CI 0.01 to 100.95)
- Dabigatran 110 mg BD (RR 0.11, 95% CI 0.00 to 4.31)
- Dabigatran 150 mg BD (RR 0.35, 95% CI 0.01 to 17.26)

There may be little or no difference in the rate of major bleeding between apixaban 10 mg and the following interventions (low‐certainty evidence; Figure 3).

- Rivaroxaban 5 mg (RR 1.06, 95% CI 0.31 to 3.64)
- Rivaroxaban 10 mg (RR 0.54, 95% CI 0.15 to 1.96)
- Rivaroxaban 15 mg (RR 0.37, 95% CI 0.07 to 2.11)
- Rivaroxaban 20 mg (RR 0.48, 95% CI 0.09 to 2.59)
- Dabigatran 50 mg BD (RR 2.55, 95% CI 0.19 to 35.05)
- Dabigatran 75 mg BD (RR 7.63, 95% CI 0.24 to 243.44)
- Dabigatran 110 mg BD (RR 0.77, 95% CI 0.08 to 7.17)
- Dabigatran 150 mg BD (RR 2.40, 95% CI 0.17 to 32.96)

There may be little or no difference in the rate of major bleeding between rivaroxaban 5 mg and the following interventions (low‐certainty evidence; Figure 3).

- Dabigatran 50 mg BD (RR 2.41, 95% CI 0.18 to 31.53)
- Dabigatran 75 mg BD (RR 7.20, 95% CI 0.23 to 221.52)
- Dabigatran 110 mg BD (RR 0.72, 95% CI 0.08 to 6.40)
- Dabigatran 150 mg BD (RR 2.26, 95% CI 0.17 to 29.65)

There may be little or no difference in the rate of major bleeding between rivaroxaban 10 mg and the following interventions (low‐certainty evidence; Figure 3).

- Dabigatran 50 mg BD (RR 4.74, 95% CI 0.35 to 64.14)
- Dabigatran 75 mg BD (RR 14.20, 95% CI 0.45 to 447.18)
- Dabigatran 110 mg BD (RR 1.42, 95% CI 0.15 to13.08)
- Dabigatran 150 mg BD (RR 4.46, 95% CI 0.33 to 60.31)

There may be little or no difference in the rate of major bleeding between rivaroxaban 15 mg and the following interventions (low‐certainty evidence; Figure 3).

- Dabigatran 50 mg BD (RR 6.85, 95% CI 0.40 to 118.00)
- Dabigatran 75 mg BD (RR 20.49, 95% CI 0.54 to 777.92)
- Dabigatran 110 mg BD (RR 2.06, 95% CI 0.17 to 24.99)
- Dabigatran 150 mg BD (RR 6.44, 95% CI 0.37 to 110.96)

There may be little or no difference in the rate of major bleeding between rivaroxaban 20 mg and the following interventions (low‐certainty evidence; Figure 3).

- Dabigatran 50 mg BD (RR 5.32, 95% CI 0.32 to 89.19)
- Dabigatran 75 mg BD (RR 15.92, 95% CI 0.43 to 591.52)
- Dabigatran 110 mg BD (RR 1.60, 95% CI 0.14 to 18.82)
- Dabigatran 150 mg BD (RR 5.00, 95% CI 0.30 to 83.87)

#### Consistency assessment

To assess the consistency of the networks, we compared the direct and indirect evidence for all treatment comparisons. No significant inconsistency was observed in our analysis. The results from the direct and indirect evidence were consistent across all included studies, providing confidence in the validity of the NMA findings.

#### Ranking of treatments (different doses of NOACs)

##### All‐cause mortality

The P scores indicate the following ranking of treatments (from lowest to highest risk of all‐cause mortality): dabigatran 110 mg BD, dabigatran 150 mg BD, rivaroxaban 15 mg, dabigatran 50 mg BD, dabigatran 75 mg BD, rivaroxaban 10 mg, rivaroxaban 20 mg, apixaban 10 mg, rivaroxaban 5 mg, placebo, and apixaban 5 mg (Table 28).

**6:** Ranking of treatments according to P values of all pairwise comparisons (different doses of non‐vitamin‐K‐antagonist oral anticoagulants versus placebo)

**Intervention**	**Rank (P value)**		
	**All‐cause mortality**	**Cardiovascular death**	**Major bleeding**
**Placebo**	10 (0.3509)	9 (0.4104)	3 (0.737)
**Apixaban 5 mg**	11 (0.1103)	11 (0.1405)	2 (0.8104)
**Apixaban 10 mg**	8 (0.3861)	7 (0.4696)	7 (0.4306)
**Rivaroxaban 5 mg**	9 (0.3623)	10 (0.3677)	6 (0.4671)
**Rivaroxaban 10 mg**	6 (0.5289)	4 (0.5551)	9 (0.2273)
**Rivaroxaban 15 mg**	3 (0.683)	3 (0.6854)	11 (0.1438)
**Rivaroxaban 20 mg**	7 (0.4168)	5 (0.4935)	10 (0.2135)
**Dabigatran 50 mg BD**	4 (0.6601)	6 (0.4757)	4 (0.6634)
**Dabigatran 75 mg BD**	5 (0.5423)	8 (0.4147)	1 (0.8241)
**Dabigatran 110 mg BD**	1 (0.7655)	2 (0.734)	8 (0.3341)
**Dabigatran 150 mg BD**	2 (0.6936)	1 (0.7535)	5 (0.6485)

##### Cardiovascular mortality

The P scores indicate the following ranking of treatments (from lowest to highest risk of cardiovascular mortality): dabigatran 150 mg BD, dabigatran 110 mg BD, rivaroxaban 15 mg, rivaroxaban 10 mg, rivaroxaban 20 mg, dabigatran 50 mg BD, apixaban 10 mg, dabigatran 75mg BD, placebo, rivaroxaban 5 mg, and apixaban 5 mg (Table 28).

##### Major bleeding

The P scores indicate the following ranking of treatments (from lowest to highest risk of major bleeding): dabigatran 75 mg BD, apixaban 5 mg, placebo, dabigatran 50 mg BD, dabigatran 150 mg BD, rivaroxaban 5 mg, apixaban 10 mg, dabigatran 110 mg BD, rivaroxaban 10 mg, rivaroxaban 20 mg, and rivaroxaban 15 (Table 28).

## Data and analyses

**Comparison 1:** NOACs (all doses combined) versus placebo: all‐cause mortality

Outcome or subgroup title	No. of studies	No. of participants	Statistical method	Effect size
1.1 All‐cause mortality (apixaban versus placebo)	2	8638	Risk Ratio (M‐H, Random, 95% CI)	1.09 [0.88, 1.35]
1.2 All‐cause mortality (rivaroxaban versus placebo)	3	21870	Risk Ratio (M‐H, Random, 95% CI)	0.82 [0.69, 0.98]
1.3 All‐cause mortality (dabigatran versus placebo)	1	1861	Risk Ratio (M‐H, Random, 95% CI)	0.57 [0.31, 1.06]

**Comparison 2:** NOACs (all doses combined) versus placebo: cardiovascular mortality

Outcome or subgroup title	No. of studies	No. of participants	Statistical method	Effect size
2.1 Cardiovascular mortality (apixaban versus placebo)	2	8638	Risk Ratio (M‐H, Random, 95% CI)	0.99 [0.77, 1.27]
2.2 Cardiovascular mortality (rivaroxaban versus placebo)	3	21870	Risk Ratio (M‐H, Random, 95% CI)	0.83 [0.69, 1.01]
2.3 Cardiovascular mortality (dabigatran versus placebo)	1	1861	Risk Ratio (M‐H, Random, 95% CI)	0.72 [0.34, 1.52]

**Comparison 3:** NOACs (all doses combined) versus placebo: major bleeding

Outcome or subgroup title	No. of studies	No. of participants	Statistical method	Effect size
3.1 Major bleeding (apixaban versus placebo)	2	8544	Risk Ratio (M‐H, Random, 95% CI)	2.41 [1.44, 4.06]
3.2 Major bleeding (rivaroxaban versus placebo)	3	21870	Risk Ratio (M‐H, Random, 95% CI)	3.31 [1.12, 9.77]
3.3 Major bleeding (dabigatran versus placebo)	1	1861	Risk Ratio (M‐H, Random, 95% CI)	1.74 [0.22, 14.12]

**Comparison 4:** Apixaban (different doses) versus placebo

Outcome or subgroup title	No. of studies	No. of participants	Statistical method	Effect size
4.1 All‐cause mortality	2		Risk Ratio (M‐H, Random, 95% CI)	Subtotals only
4.1.1 Apixaban 5 mg versus placebo	1	928	Risk Ratio (M‐H, Random, 95% CI)	1.77 [0.79, 3.96]
4.1.2 Apixaban 10 mg versus placebo	2	8321	Risk Ratio (M‐H, Random, 95% CI)	1.06 [0.86, 1.32]
4.1.3 Apixaban total versus placebo	2	8638	Risk Ratio (M‐H, Random, 95% CI)	1.09 [0.88, 1.35]
4.2 Cardiovascular mortality	2		Risk Ratio (M‐H, Random, 95% CI)	Subtotals only
4.2.1 Apixaban 5 mg versus placebo	1	928	Risk Ratio (M‐H, Random, 95% CI)	1.93 [0.84, 4.40]
4.2.2 Apixaban 10 mg versus placebo	2	8321	Risk Ratio (M‐H, Random, 95% CI)	0.94 [0.73, 1.22]
4.2.3 Apixaban total versus placebo	2	8638	Risk Ratio (M‐H, Random, 95% CI)	0.99 [0.77, 1.27]
4.3 Major bleeding	2		Risk Ratio (M‐H, Random, 95% CI)	Subtotals only
4.3.1 Apixaban 5 mg versus placebo	1	914	Risk Ratio (M‐H, Random, 95% CI)	0.38 [0.02, 7.89]
4.3.2 Apixaban 10 mg versus placebo	2	8229	Risk Ratio (M‐H, Random, 95% CI)	2.56 [1.52, 4.30]
4.3.3 Apixaban total versus placebo	2	8544	Risk Ratio (M‐H, Random, 95% CI)	2.41 [1.44, 4.06]
4.4 Myocardial infarction	2		Risk Ratio (M‐H, Random, 95% CI)	Subtotals only
4.4.1 Apixaban 5 mg versus placebo	1	928	Risk Ratio (M‐H, Random, 95% CI)	0.67 [0.29, 1.58]
4.4.2 Apixaban 10 mg versus placebo	2	8321	Risk Ratio (M‐H, Random, 95% CI)	0.90 [0.71, 1.14]
4.4.3 Apixaban total versus placebo	2	8638	Risk Ratio (M‐H, Random, 95% CI)	0.88 [0.67, 1.16]
4.5 Stroke	2		Risk Ratio (M‐H, Random, 95% CI)	Subtotals only
4.5.1 Apixaban 5 mg versus placebo	1	928	Risk Ratio (M‐H, Random, 95% CI)	0.38 [0.02, 7.99]
4.5.2 Apixaban 10 mg versus placebo	2	8321	Risk Ratio (M‐H, Random, 95% CI)	0.68 [0.41, 1.15]
4.5.3 Apixaban total versus placebo	2	8638	Risk Ratio (M‐H, Random, 95% CI)	0.66 [0.40, 1.11]
4.6 Stent thrombosis	1	7392	Risk Ratio (M‐H, Random, 95% CI)	0.73 [0.47, 1.12]
4.6.1 Apixaban 10 mg versus placebo	1	7392	Risk Ratio (M‐H, Random, 95% CI)	0.73 [0.47, 1.12]
4.7 Non‐major bleeding	2		Risk Ratio (M‐H, Random, 95% CI)	Subtotals only
4.7.1 Apixaban 5 mg versus placebo	1	914	Risk Ratio (M‐H, Random, 95% CI)	1.90 [0.39, 9.37]
4.7.2 apixaban 10mg versus placebo	2	8229	Risk Ratio (M‐H, Random, 95% CI)	2.74 [1.45, 5.17]
4.7.3 apixaban total versus placebo	2	8544	Risk Ratio (M‐H, Random, 95% CI)	2.71 [1.47, 5.01]

**Comparison 5:** Rivaroxaban (different doses) versus placebo

Outcome or subgroup title	No. of studies	No. of participants	Statistical method	Effect size
5.1 All‐cause mortality	3		Risk Ratio (M‐H, Random, 95% CI)	Subtotals only
5.1.1 Rivaroxaban 5 mg versus placebo	3	14732	Risk Ratio (M‐H, Random, 95% CI)	1.00 [0.55, 1.82]
5.1.2 Rivaroxaban 10 mg versus placebo	2	12444	Risk Ratio (M‐H, Random, 95% CI)	0.84 [0.56, 1.26]
5.1.3 Rivaroxaban 15 mg versus placebo	1	1516	Risk Ratio (M‐H, Random, 95% CI)	0.61 [0.18, 2.08]
5.1.4 Rivaroxaban 20 mg versus placebo	1	1771	Risk Ratio (M‐H, Random, 95% CI)	1.07 [0.47, 2.40]
5.1.5 Rivaroxaban total versus placebo	3	21870	Risk Ratio (M‐H, Random, 95% CI)	0.82 [0.69, 0.98]
5.2 Cardiovascular mortality	3		Risk Ratio (M‐H, Random, 95% CI)	Subtotals only
5.2.1 Rivaroxaban 5 mg versus placebo	3	14732	Risk Ratio (M‐H, Random, 95% CI)	1.14 [0.53, 2.44]
5.2.2 Rivaroxaban 10 mg versus placebo	2	12444	Risk Ratio (M‐H, Random, 95% CI)	0.90 [0.72, 1.13]
5.2.3 Rivaroxaban 15 mg versus placebo	1	1516	Risk Ratio (M‐H, Random, 95% CI)	0.75 [0.22, 2.62]
5.2.4 Rivaroxaban 20 mg versus placebo	1	1771	Risk Ratio (M‐H, Random, 95% CI)	1.17 [0.49, 2.80]
5.2.5 Rivaroxaban total versus placebo	3	21870	Risk Ratio (M‐H, Random, 95% CI)	0.83 [0.69, 1.01]
5.3 Major bleeding	3		Risk Ratio (M‐H, Random, 95% CI)	Subtotals only
5.3.1 Rivaroxaban 5 mg versus placebo	3	14732	Risk Ratio (M‐H, Random, 95% CI)	2.39 [1.11, 5.16]
5.3.2 Rivaroxaban 10 mg versus placebo	2	12444	Risk Ratio (M‐H, Random, 95% CI)	6.17 [1.83, 20.85]
5.3.3 Rivaroxaban 15 mg versus placebo	1	1516	Risk Ratio (M‐H, Random, 95% CI)	19.55 [2.36, 161.85]
5.3.4 Rivaroxaban 20 mg versus placebo	1	1771	Risk Ratio (M‐H, Random, 95% CI)	15.19 [1.90, 121.15]
5.3.5 Rivaroxaban total versus placebo	3	21870	Risk Ratio (M‐H, Random, 95% CI)	3.31 [1.12, 9.77]
5.4 Myocardial infarction	3		Risk Ratio (M‐H, Random, 95% CI)	Subtotals only
5.4.1 Rivaroxaban 5 mg versus placebo	3	14732	Risk Ratio (M‐H, Random, 95% CI)	0.95 [0.81, 1.11]
5.4.2 Rivaroxaban 10 mg versus placebo	2	12444	Risk Ratio (M‐H, Random, 95% CI)	0.77 [0.65, 0.92]
5.4.3 Rivaroxaban 15 mg versus placebo	1	1516	Risk Ratio (M‐H, Random, 95% CI)	1.11 [0.64, 1.93]
5.4.4 Rivaroxaban 20 mg versus placebo	1	1771	Risk Ratio (M‐H, Random, 95% CI)	0.69 [0.40, 1.19]
5.4.5 Rivaroxaban total versus placebo	3	21870	Risk Ratio (M‐H, Random, 95% CI)	0.88 [0.75, 1.03]
5.5 Stroke	3		Risk Ratio (M‐H, Random, 95% CI)	Subtotals only
5.5.1 Rivaroxaban 5 mg versus placebo	3	14732	Risk Ratio (M‐H, Random, 95% CI)	0.99 [0.68, 1.44]
5.5.2 Rivaroxaban 10 mg versus placebo	2	12444	Risk Ratio (M‐H, Random, 95% CI)	1.25 [0.85, 1.83]
5.5.3 Rivaroxaban 15 mg versus placebo	1	1516	Risk Ratio (M‐H, Random, 95% CI)	0.25 [0.01, 4.43]
5.5.4 Rivaroxaban 20 mg versus placebo	1	1771	Risk Ratio (M‐H, Random, 95% CI)	0.32 [0.04, 2.62]
5.5.5 Rivaroxaban total versus placebo	3	21870	Risk Ratio (M‐H, Random, 95% CI)	0.84 [0.45, 1.55]
5.6 Stent thrombosis	2		Risk Ratio (M‐H, Random, 95% CI)	Subtotals only
5.6.1 Rivaroxaban 5 mg versus placebo	2	13264	Risk Ratio (M‐H, Random, 95% CI)	0.76 [0.49, 1.19]
5.6.2 Rivaroxaban 10 mg versus placebo	1	10228	Risk Ratio (M‐H, Random, 95% CI)	0.71 [0.50, 1.01]
5.6.3 Rivaroxaban total versus placebo	2	18379	Risk Ratio (M‐H, Random, 95% CI)	0.76 [0.52, 1.12]
5.7 Non‐major bleeding	3		Risk Ratio (M‐H, Random, 95% CI)	Subtotals only
5.7.1 Rivaroxaban 5 mg versus placebo	3	14732	Risk Ratio (M‐H, Random, 95% CI)	1.71 [1.04, 2.80]
5.7.2 Rivaroxaban 10 mg versus placebo	2	12444	Risk Ratio (M‐H, Random, 95% CI)	2.52 [1.54, 4.13]
5.7.3 Rivaroxaban 15 mg versus placebo	1	1516	Risk Ratio (M‐H, Random, 95% CI)	6.52 [1.20, 35.43]
5.7.4 Rivaroxaban 20 mg versus placebo	1	1771	Risk Ratio (M‐H, Random, 95% CI)	4.75 [0.92, 24.39]
5.7.5 Rivaroxaban total versus placebo	3	21870	Risk Ratio (M‐H, Random, 95% CI)	2.18 [1.41, 3.35]
5.8 Systemic embolism	1		Risk Ratio (M‐H, Random, 95% CI)	Subtotals only
5.8.1 Rivaroxaban 5 mg versus placebo	1	1468	Risk Ratio (M‐H, Random, 95% CI)	0.54 [0.03, 10.36]
5.8.2 Rivaroxaban 10 mg versus placebo	1	2216	Risk Ratio (M‐H, Random, 95% CI)	0.16 [0.01, 3.03]
5.8.3 Rivaroxaban 15 mg versus placebo	1	1516	Risk Ratio (M‐H, Random, 95% CI)	0.46 [0.02, 8.97]
5.8.4 Rivaroxaban 20 mg versus placebo	1	1771	Risk Ratio (M‐H, Random, 95% CI)	0.27 [0.01, 5.24]
5.8.5 Rivaroxaban total versus placebo	1	3491	Risk Ratio (M‐H, Random, 95% CI)	0.07 [0.00, 1.38]

**Comparison 6:** Dabigatran (different doses) versus placebo

Outcome or subgroup title	No. of studies	No. of participants	Statistical method	Effect size
6.1 All‐cause mortality	1		Risk Ratio (M‐H, Random, 95% CI)	Subtotals only
6.1.1 Dabigatran 50 mg BD versus placebo	1	740	Risk Ratio (M‐H, Random, 95% CI)	0.57 [0.24, 1.35]
6.1.2 Dabigatran 75 mg BD versus placebo	1	739	Risk Ratio (M‐H, Random, 95% CI)	0.72 [0.32, 1.60]
6.1.3 Dabigatran 110 mg BD versus placebo	1	777	Risk Ratio (M‐H, Random, 95% CI)	0.46 [0.19, 1.12]
6.1.4 Dabigatran 150 mg BD versus placebo	1	718	Risk Ratio (M‐H, Random, 95% CI)	0.53 [0.22, 1.31]
6.1.5 Dabigatran total versus placebo	1	1861	Risk Ratio (M‐H, Random, 95% CI)	0.57 [0.31, 1.06]
6.2 Cardiovascular mortality	1		Risk Ratio (M‐H, Random, 95% CI)	Subtotals only
6.2.1 Dabigatran 50 mg BD versus placebo	1	740	Risk Ratio (M‐H, Random, 95% CI)	0.89 [0.35, 2.29]
6.2.2 Dabigatran 75 mg BD versus placebo	1	739	Risk Ratio (M‐H, Random, 95% CI)	1.01 [0.40, 2.51]
6.2.3 Dabigatran 110 mg BD versus placebo	1	777	Risk Ratio (M‐H, Random, 95% CI)	0.51 [0.17, 1.50]
6.2.4 Dabigatran 150 mg BD versus placebo	1	718	Risk Ratio (M‐H, Random, 95% CI)	0.48 [0.15, 1.53]
6.2.5 Dabigatran total versus placebo	1	1861	Risk Ratio (M‐H, Random, 95% CI)	0.72 [0.34, 1.52]
6.3 Major bleeding	1		Risk Ratio (M‐H, Random, 95% CI)	Subtotals only
6.3.1 Dabigatran 50 mg BD versus placebo	1	740	Risk Ratio (M‐H, Random, 95% CI)	1.01 [0.06, 16.01]
6.3.2 Dabigatran 75 mg BD versus placebo	1	739	Risk Ratio (M‐H, Random, 95% CI)	0.34 [0.01, 8.22]
6.3.3 Dabigatran 110 mg BD versus placebo	1	777	Risk Ratio (M‐H, Random, 95% CI)	4.57 [0.54, 38.93]
6.3.4 Dabigatran 150 mg BD versus placebo	1	718	Risk Ratio (M‐H, Random, 95% CI)	1.07 [0.07, 17.03]
6.3.5 Dabigatran total versus placebo	1	1861	Risk Ratio (M‐H, Random, 95% CI)	1.74 [0.22, 14.12]
6.4 Myocardial infarction	1		Risk Ratio (M‐H, Random, 95% CI)	Subtotals only
6.4.1 Dabigatran 50 mg BD versus placebo	1	740	Risk Ratio (M‐H, Random, 95% CI)	2.26 [0.70, 7.28]
6.4.2 Dabigatran 75 mg BD versus placebo	1	739	Risk Ratio (M‐H, Random, 95% CI)	2.02 [0.61, 6.64]
6.4.3 Dabigatran 110 mg BD versus placebo	1	777	Risk Ratio (M‐H, Random, 95% CI)	1.60 [0.47, 5.42]
6.4.4 Dabigatran 150 mg BD versus placebo	1	718	Risk Ratio (M‐H, Random, 95% CI)	2.14 [0.65, 7.04]
6.4.5 Dabigatran total versus placebo	1	1861	Risk Ratio (M‐H, Random, 95% CI)	1.99 [0.71, 5.60]
6.5 Stroke	1		Risk Ratio (M‐H, Random, 95% CI)	Subtotals only
6.5.1 Dabigatran 50 mg BD versus placebo	1	740	Risk Ratio (M‐H, Random, 95% CI)	0.14 [0.01, 2.77]
6.5.2 Dabigatran 75 mg BD versus placebo	1	739	Risk Ratio (M‐H, Random, 95% CI)	0.34 [0.04, 3.22]
6.5.3 Dabigatran 110 mg BD versus placebo	1	777	Risk Ratio (M‐H, Random, 95% CI)	0.13 [0.01, 2.52]
6.5.4 Dabigatran 150 mg BD versus placebo	1	718	Risk Ratio (M‐H, Random, 95% CI)	0.15 [0.01, 2.95]
6.5.5 Dabigatran versus placebo	1	1861	Risk Ratio (M‐H, Random, 95% CI)	0.08 [0.01, 0.80]

**Comparison 7:** Apixaban 5 mg versus apixaban 10 mg

Outcome or subgroup title	No. of studies	No. of participants	Statistical method	Effect size
7.1 All‐cause mortality	1	635	Risk Ratio (M‐H, Random, 95% CI)	2.21 [0.78, 6.28]
7.2 Cardiovascular mortality	1	635	Risk Ratio (M‐H, Random, 95% CI)	2.76 [0.89, 8.57]
7.3 Major bleeding	1	630	Risk Ratio (M‐H, Random, 95% CI)	0.14 [0.01, 2.75]
7.4 Myocardial infarction	1	635	Risk Ratio (M‐H, Random, 95% CI)	1.17 [0.40, 3.44]
7.5 Stroke	1	635	Risk Ratio (M‐H, Random, 95% CI)	0.33 [0.01, 8.18]
7.6 Non‐major bleeding	1	630	Risk Ratio (M‐H, Random, 95% CI)	1.50 [0.25, 8.92]

**Comparison 8:** Rivaroxaban 5 mg versus rivaroxaban 10 mg

Outcome or subgroup title	No. of studies	No. of participants	Statistical method	Effect size
8.1 All‐cause mortality	2	11593	Risk Ratio (M‐H, Random, 95% CI)	1.57 [0.31, 8.03]
8.2 Cardiovascular mortality	2	11593	Risk Ratio (M‐H, Random, 95% CI)	1.56 [0.30, 8.11]
8.3 Major bleeding	2	11593	Risk Ratio (M‐H, Random, 95% CI)	0.61 [0.21, 1.72]
8.4 Myocardial infarction	2	11593	Risk Ratio (M‐H, Random, 95% CI)	1.17 [0.97, 1.41]
8.5 Stroke	2	11593	Risk Ratio (M‐H, Random, 95% CI)	0.85 [0.58, 1.25]
8.6 Stent thrombosis	1	10229	Risk Ratio (M‐H, Random, 95% CI)	0.92 [0.62, 1.37]
8.7 Non‐major bleeding	2	11593	Risk Ratio (M‐H, Random, 95% CI)	0.65 [0.42, 1.00]

**Comparison 9:** Rivaroxaban 5 mg versus rivaroxaban 15 mg

Outcome or subgroup title	No. of studies	No. of participants	Statistical method	Effect size
9.1 All‐cause mortality	1	664	Risk Ratio (M‐H, Random, 95% CI)	3.47 [0.95, 12.69]
9.2 Cardiovascular mortality	1	664	Risk Ratio (M‐H, Random, 95% CI)	3.47 [0.95, 12.69]
9.3 Major bleeding	1	664	Risk Ratio (M‐H, Random, 95% CI)	0.19 [0.02, 1.59]
9.4 Myocardial infarction	1	664	Risk Ratio (M‐H, Random, 95% CI)	0.94 [0.46, 1.92]
9.5 Stroke	1	664	Risk Ratio (M‐H, Random, 95% CI)	3.47 [0.14, 84.77]
9.6 Non‐major bleeding	1	664	Risk Ratio (M‐H, Random, 95% CI)	0.29 [0.03, 2.57]

**Comparison 10:** Rivaroxaban 5 mg versus rivaroxaban 20 mg

Outcome or subgroup title	No. of studies	No. of participants	Statistical method	Effect size
10.1 All‐cause mortality	1	919	Risk Ratio (M‐H, Random, 95% CI)	1.98 [0.80, 4.95]
10.2 Cardiovascular mortality	1	919	Risk Ratio (M‐H, Random, 95% CI)	2.23 [0.87, 5.73]
10.3 Major bleeding	1	919	Risk Ratio (M‐H, Random, 95% CI)	0.25 [0.03, 1.97]
10.4 Myocardial infarction	1	919	Risk Ratio (M‐H, Random, 95% CI)	1.52 [0.75, 3.08]
10.5 Stroke	1	919	Risk Ratio (M‐H, Random, 95% CI)	1.98 [0.12, 31.61]
10.6 Non‐major bleeding	1	919	Risk Ratio (M‐H, Random, 95% CI)	0.40 [0.05, 3.38]

**Comparison 11:** Rivaroxaban 10 mg versus rivaroxaban 15 mg

Outcome or subgroup title	No. of studies	No. of participants	Statistical method	Effect size
11.1 All‐cause mortality	1	1412	Risk Ratio (M‐H, Random, 95% CI)	0.90 [0.24, 3.37]
11.2 Cardiovascular mortality	1	1412	Risk Ratio (M‐H, Random, 95% CI)	0.90 [0.24, 3.37]
11.3 Major bleeding	1	1412	Risk Ratio (M‐H, Random, 95% CI)	0.90 [0.35, 2.28]
11.4 Myocardial infarction	1	1412	Risk Ratio (M‐H, Random, 95% CI)	0.65 [0.36, 1.18]
11.5 Stroke	1	1412	Risk Ratio (M‐H, Random, 95% CI)	3.04 [0.16, 56.32]
11.6 Non‐major bleeding	1	1412	Risk Ratio (M‐H, Random, 95% CI)	0.51 [0.14, 1.78]

**Comparison 12:** Rivaroxaban 10 mg versus rivaroxaban 20 mg

Outcome or subgroup title	No. of studies	No. of participants	Statistical method	Effect size
12.1 All‐cause mortality	1	1667	Risk Ratio (M‐H, Random, 95% CI)	0.51 [0.20, 1.33]
12.2 Cardiovascular mortality	1	1667	Risk Ratio (M‐H, Random, 95% CI)	0.58 [0.22, 1.53]
12.3 Major bleeding	1	1667	Risk Ratio (M‐H, Random, 95% CI)	1.16 [0.50, 2.69]
12.4 Myocardial infarction	1	1667	Risk Ratio (M‐H, Random, 95% CI)	1.06 [0.59, 1.89]
12.5 Stroke	1	1667	Risk Ratio (M‐H, Random, 95% CI)	2.31 [0.26, 20.66]
12.6 Non‐major bleeding	1	1667	Risk Ratio (M‐H, Random, 95% CI)	0.69 [0.21, 2.27]

**Comparison 13:** Rivaroxaban 15 mg versus rivaroxaban 20 mg

Outcome or subgroup title	No. of studies	No. of participants	Statistical method	Effect size
13.1 All‐cause mortality	1	967	Risk Ratio (M‐H, Random, 95% CI)	0.57 [0.16, 2.10]
13.2 Cardiovascular mortality	1	967	Risk Ratio (M‐H, Random, 95% CI)	0.64 [0.17, 2.41]
13.3 Major bleeding	1	967	Risk Ratio (M‐H, Random, 95% CI)	1.29 [0.45, 3.68]
13.4 Myocardial infarction	1	967	Risk Ratio (M‐H, Random, 95% CI)	1.62 [0.83, 3.16]
13.5 Stroke	1	967	Risk Ratio (M‐H, Random, 95% CI)	0.57 [0.02, 13.99]
13.6 Non‐major bleeding	1	967	Risk Ratio (M‐H, Random, 95% CI)	1.37 [0.37, 5.08]

**Comparison 14:** Dabigatran 50 mg BD versus dabigatran 75 mg BD

Outcome or subgroup title	No. of studies	No. of participants	Statistical method	Effect size
14.1 All‐cause mortality	1	737	Risk Ratio (M‐H, Random, 95% CI)	0.80 [0.32, 2.00]
14.2 Cardiovascular mortality	1	737	Risk Ratio (M‐H, Random, 95% CI)	0.89 [0.35, 2.27]
14.3 Major bleeding	1	737	Risk Ratio (M‐H, Random, 95% CI)	2.99 [0.12, 73.21]
14.4 Myocardial infarction	1	737	Risk Ratio (M‐H, Random, 95% CI)	1.12 [0.44, 2.88]
14.5 Stroke	1	737	Risk Ratio (M‐H, Random, 95% CI)	0.33 [0.01, 8.13]

**Comparison 15:** Dabigatran 50 mg BD versus dabigatran 110 mg BD

Outcome or subgroup title	No. of studies	No. of participants	Statistical method	Effect size
15.1 All‐cause mortality	1	775	Risk Ratio (M‐H, Random, 95% CI)	1.26 [0.46, 3.43]
15.2 Cardiovascular mortality	1	775	Risk Ratio (M‐H, Random, 95% CI)	1.76 [0.58, 5.33]
15.3 Major bleeding	1	775	Risk Ratio (M‐H, Random, 95% CI)	0.22 [0.03, 1.87]
15.4 Myocardial infarction	1	775	Risk Ratio (M‐H, Random, 95% CI)	1.41 [0.53, 3.76]
15.5 Stroke	1	775	Risk Ratio (M‐H, Random, 95% CI)	Not estimable

**Comparison 16:** Dabigatran 50 mg BD versus dabigatran 150 mg BD

Outcome or subgroup title	No. of studies	No. of participants	Statistical method	Effect size
16.1 All‐cause mortality	1	716	Risk Ratio (M‐H, Random, 95% CI)	1.07 [0.39, 2.93]
16.2 Cardiovascular mortality	1	716	Risk Ratio (M‐H, Random, 95% CI)	1.88 [0.57, 6.19]
16.3 Major bleeding	1	716	Risk Ratio (M‐H, Random, 95% CI)	0.94 [0.06, 14.98]
16.4 Myocardial infarction	1	716	Risk Ratio (M‐H, Random, 95% CI)	1.06 [0.41, 2.71]
16.5 Stroke	1	716	Risk Ratio (M‐H, Random, 95% CI)	Not estimable

**Comparison 17:** Dabigatran 75 mg BD versus dabigatran 110 mg BD

Outcome or subgroup title	No. of studies	No. of participants	Statistical method	Effect size
17.1 All‐cause mortality	1	774	Risk Ratio (M‐H, Random, 95% CI)	1.58 [0.61, 4.10]
17.2 Cardiovascular mortality	1	774	Risk Ratio (M‐H, Random, 95% CI)	1.99 [0.67, 5.87]
17.3 Major bleeding	1	774	Risk Ratio (M‐H, Random, 95% CI)	0.10 [0.01, 1.81]
17.4 Myocardial infarction	1	774	Risk Ratio (M‐H, Random, 95% CI)	1.26 [0.46, 3.44]
17.5 Stroke	1	774	Risk Ratio (M‐H, Random, 95% CI)	3.31 [0.14, 80.97]

**Comparison 18:** Dabigatran 75 mg BD versus dabigatran 150 mg BD

Outcome or subgroup title	No. of studies	No. of participants	Statistical method	Effect size
18.1 All‐cause mortality	1	715	Risk Ratio (M‐H, Random, 95% CI)	1.35 [0.52, 3.50]
18.2 Cardiovascular mortality	1	715	Risk Ratio (M‐H, Random, 95% CI)	2.12 [0.66, 6.83]
18.3 Major bleeding	1	715	Risk Ratio (M‐H, Random, 95% CI)	0.31 [0.01, 7.69]
18.4 Myocardial infarction	1	715	Risk Ratio (M‐H, Random, 95% CI)	0.94 [0.36, 2.48]
18.5 Stroke	1	715	Risk Ratio (M‐H, Random, 95% CI)	2.83 [0.12, 69.22]

**Comparison 19:** Dabigatran 110 mg BD versus dabigatran 150 mg BD

Outcome or subgroup title	No. of studies	No. of participants	Statistical method	Effect size
19.1 All‐cause mortality	1	753	Risk Ratio (M‐H, Random, 95% CI)	0.85 [0.30, 2.41]
19.2 Cardiovascular mortality	1	753	Risk Ratio (M‐H, Random, 95% CI)	1.07 [0.29, 3.95]
19.3 Major bleeding	1	753	Risk Ratio (M‐H, Random, 95% CI)	4.27 [0.50, 36.40]
19.4 Myocardial infarction	1	753	Risk Ratio (M‐H, Random, 95% CI)	0.75 [0.27, 2.04]
19.5 Stroke	1	753	Risk Ratio (M‐H, Random, 95% CI)	Not estimable

## Characteristics of studies

### Characteristics of included studies [ordered by study ID]

**Table:** APPRAISE 1

***Study characteristics***		
Methods	Randomised, double‐blind, placebo‐controlled trial Study dates: May 2006–October 2007	
Participants	Setting: 151 sites in 14 countries in Europe, the Middle East, and North America Number randomised/analysed: 1715/1715 Age: median 61 years Sex (male/female): 77%/23%	
Interventions	Experimental group 1 (n = 315): apixaban 2.5 mg twice daily Experimental group 2 (n= 315): apixaban 10mg once daily Control group (n = 599): placebo Cointerventions: all participants received aspirin, and 76% received additional clopidogrel. Inclusion criteria Age 18–90 years Recent (within 7 days) STE‐ACS or NSTE‐ACS Clinical stability with evidence‐based care ≥ 1 additional risk factor for recurrent ischaemic events Exclusion criteria Aspirin allergy Planned catheterisation, percutaneous coronary intervention, coronary bypass surgery, or other invasive procedure Persistent severe hypertension Severe renal insufficiency Active bleeding or a high risk for bleeding Coagulopathy Acute pericarditis or pericardial effusion Stroke within 3 months New York Heart Association class IV heart failure Thrombocytopenia Anaemia Indication for ongoing anticoagulation Long‐term nonsteroidal anti‐inflammatory drug or high‐dose aspirin use Ongoing treatment with strong CYP3A4 inhibitors Participation in an investigational drug or device trial within 30 days Women of childbearing potential	
Outcomes	Primary outcomes ISTH major or clinically relevant non‐major bleeding (event rate was number of participants with events divided by the number of participants treated, measured as a percentage) Secondary outcomes Cardiovascular death Myocardial infarction Severe recurrent ischaemia Ischaemic stroke	
Notes	Sponsor: Bristol‐Myers Squibb	
***Risk of bias***		
**Bias**	**Authors' judgement**	**Support for judgement**
Random sequence generation (selection bias)	Low risk	Randomised via a centralised interactive voice response system.
Allocation concealment (selection bias)	Low risk	Randomised via a centralised interactive voice response system.
Blinding of participants and personnel (performance bias) All outcomes	Low risk	Double‐blind study.
Blinding of outcome assessment (detection bias) All outcomes	Low risk	Outcomes were reviewed by a blinded independent clinical events committee.
Incomplete outcome data (attrition bias) All outcomes	Low risk	Cleary described in Figure 1. Loss to follow‐up ~10%.
Selective reporting (reporting bias)	Low risk	All outcomes in the trial registration reported. Registered in ClinicalTrials.gov (NCT00313300).
Other bias	Low risk	The clinical trial data were managed at Bristol‐Myers Squibb, but the locked database was transferred in full to the Duke Clinical Research Institute for analysis. All analyses were performed independently by statisticians at the Duke Clinical Research Institute. The sponsors reviewed and commented on the manuscript, but their approval was not required.

**Table:** APPRAISE 2

***Study characteristics***		
Methods	Randomised, double‐blind, placebo‐controlled trial Study dates: March 2009–November 2010	
Participants	Setting: 858 sites in 39 countries Number randomised/analysed: 7392/7392 Age: median 67 years Sex (male/female): 67%/33%	
Interventions	Experimental group (n = 3705): apixaban 5 mg twice daily Control group (n = 3687): placebo, twice daily Cointerventions: all participants received aspirin, and 81% received additional clopidogrel. Inclusion criteria ACS (myocardial infarction, with or without ST‐segment elevation, or unstable angina) within the previous 7 days Symptoms of myocardial ischaemia lasting ≥ 10 minutes with the person at rest Either elevated levels of cardiac biomarkers or dynamic ST‐segment depression or elevation of ≥ 0.1 mV Clinical stability with standard treatment (e.g. aspirin or aspirin plus any P2Y12‐receptor antagonist) ≥ 2 of the following high‐risk characteristics: age ≥ 65 years, diabetes mellitus, myocardial infarction within the previous 5 years, cerebrovascular disease, peripheral vascular disease, clinical heart failure or a LVEF < 40% in association with the index event, impaired renal function with a calculated creatinine clearance < 60 mL/minute, and no revascularisation after the index event Exclusion criteria Severe hypertension Active bleeding or high risk for major bleeding Haemoglobin < 9 g/day	
Outcomes	Primary Outcomes Efficacy: cardiovascular death/myocardial infarction/ischaemic stroke Safety: major bleeding using TIMI criteria Secondary Outcomes Efficacy Cardiovascular death/myocardial infarction/ischaemic stroke/unstable angina Cardiovascular death/myocardial infarction/ischaemic or haemorrhagic stroke/fatal bleeding Cardiovascular death/myocardial infarction/ischaemic stroke or haemorrhagic stroke Cardiovascular death Myocardial infarction Stroke Stent thrombosis Safety Major or minor bleeding using TIMI criteria ISTH bleeding GUSTO bleeding	
Notes	Supported by Bristol‐Myers Squibb and Pfizer.	
***Risk of bias***		
**Bias**	**Authors' judgement**	**Support for judgement**
Random sequence generation (selection bias)	Low risk	Quote: "Participants were randomly assigned, in a 1:1 ratio, to receive apixaban, at a dose of 5 mg BD, or matching placebo".
Allocation concealment (selection bias)	Low risk	Quote: "Randomisation was performed in a blinded fashion with the use of an interactive voice‐response system, in permuted blocks of two, stratified according to site and according to planned long‐term use of aspirin or aspirin plus a P2Y12‐receptor antagonist".
Blinding of participants and personnel (performance bias) All outcomes	Low risk	Double‐blind study.
Blinding of outcome assessment (detection bias) All outcomes	Low risk	The primary and secondary outcomes were adjudicated with the use of prespecified criteria by an independent clinical events committee.
Incomplete outcome data (attrition bias) All outcomes	Low risk	Among the participants who underwent randomisation, 81 (1.1%) withdrew consent and 50 (0.7%) were lost to follow‐up for the primary outcome during the intended treatment period.
Selective reporting (reporting bias)	Low risk	All outcomes were defined a priori in the published protocol. The trial protocol and statistical analysis plan are available at NEJM.org.
Other bias	Low risk	Sponsors (Bristol‐Myers Squibb and Pfizer). Sponsor approval not required.

**Table:** ATLAS ACS

***Study characteristics***		
Methods	Randomised, double‐blind, placebo‐controlled trial Study dates: November 2006–October 2008	
Participants	Setting: 297 sites in 27 countries Number randomised/analysed: 3491/3491 Age: median 57 years Sex (male/female): 78%/22%	
Interventions	Experimental group (n = 2331): rivaroxaban 5 mg once daily, 10 mg once daily, 15 mg once daily, 20 mg once daily Control group (n = 1160): placebo Cointerventions: all participants received aspirin and 80% received additional clopidogrel. Inclusion Criteria Symptoms suggestive of ACS lasting ≥ 10 minutes at rest occurring within 7 days of randomisation Have a diagnosis of STEMI or NSTEMI/unstable angina (i.e. chest pain or discomfort) with ≥ 1 protocol‐defined high risk feature Exclusion Criteria Active bleeding or high risk of bleeding or intracranial haemorrhage (bleeding within the skull enclosing the brain) Need for continued anticoagulant therapy Significantly impaired renal or hepatic function Severe concomitant diseases (e.g. cardiogenic shock (heart damage that results in insufficient blood supply to other parts or organs of the body), refractory ventricular arrhythmias (irregular contractions of the heart unresponsive to treatment), or any severe condition that would limit life expectancy of the patient to < 6 months)	
Outcomes	Primary outcomes TIMI clinically significant bleeding events Composite endpoint of all‐cause death, myocardial infarction, stroke (ischaemic, haemorrhagic or unknown), or severe recurrent ischaemia requiring revascularisation) Secondary outcomes Composite endpoint of all‐cause death, myocardial infarction, or stroke Number of deaths (all‐cause) Composite endpoint of all‐cause death, myocardial infarction, stroke, severe recurrent ischemia requiring revascularisation, and TIMI major or minor bleeding, to assess the net clinical benefit	
Notes	Funding: Johnson & Johnson Pharmaceutical Research & Development and Bayer Healthcare AG	
***Risk of bias***		
**Bias**	**Authors' judgement**	**Support for judgement**
Random sequence generation (selection bias)	Low risk	Participants were randomised with a block randomisation method at 1:1:1.
Allocation concealment (selection bias)	Low risk	Block randomisation.
Blinding of participants and personnel (performance bias) All outcomes	Low risk	Investigators and participants were blinded to treatment.
Blinding of outcome assessment (detection bias) All outcomes	Low risk	A blinded clinical events committee adjudicated all the components of the main safety and efficacy outcomes.
Incomplete outcome data (attrition bias) All outcomes	Low risk	The primary analysis was based on the ITT population, including all participants who were randomly assigned to a treatment group, irrespective of administration. "Figure 2 Trial profile" shows causes of drug discontinuation and details of ITT analysis.
Selective reporting (reporting bias)	Low risk	All prespecified outcomes were reported. This study is registered at ClinicalTrials.gov (NCT00402597).
Other bias	Low risk	This study was designed as a collaboration between the TIMI Study Group, the sponsors, and a steering committee of investigators. All analyses were undertaken by the TIMI Study Group, with an independent copy of the complete database.

**Table:** ATLAS ACS 2

***Study characteristics***		
Methods	Randomised, double‐blind, placebo‐controlled trial Study dates: November 2008–September 2011	
Participants	Setting: 766 sites in 44 countries Number randomised/analysed: 15526/15526 Age: median 62 years Sex (male/female): 75%/25%	
Interventions	Experimental group 1 (n = 5174): rivaroxaban 2.5 mg, 1 tablet twice daily Experimental group 2 (n = 5176): rivaroxaban 5 mg, 1 tablet twice daily Control group (n = 5176): placebo, 1 tablet twice daily Cointerventions: all participants received aspirin, and 93% received additional clopidogrel. Inclusion criteria Current aspirin therapy alone or in combination with a thienopyridine as per national or local dosing recommendation Hospitalisation for symptoms suggestive of ACS that lasted ≥ 10 minutes at rest and occurred ≤ 48 hours before going to the hospital Exclusion criteria Any condition that, in the opinion of the investigator, contraindicates anticoagulant therapy or would have an unacceptable risk of bleeding Need for continued anticoagulant therapy Significant renal impairment or known significant liver disease	
Outcomes	Primary outcomes Percentage of participants with the composite endpoint of cardiovascular death, myocardial infarction, or stroke Secondary outcomes Percentage of participants with the composite endpoint of all‐cause death, myocardial infarction, or stroke Percentage of participants with the composite endpoint of cardiovascular death, myocardial infarction, ischemic stroke, or TIMI major bleeding event not associated with coronary artery bypass graft surgery Percentage of participants with the composite endpoint of cardiovascular death, myocardial infarction, stroke, or severe recurrent ischaemia requiring revascularisation Percentage of participants with the composite endpoint of cardiovascular death, myocardial infarction, stroke, or severe recurrent ischaemia leading to hospitalisation	
Notes	Sponsors: Johnson & Johnson Pharmaceutical Research & Development, L.L.C and Bayer	
***Risk of bias***		
**Bias**	**Authors' judgement**	**Support for judgement**
Random sequence generation (selection bias)	Low risk	Quote: "Participants were randomly assigned in a 1:1:1 fashion to twice‐daily administration of either 2.5 mg or 5.0 mg of rivaroxaban or placebo".
Allocation concealment (selection bias)	Low risk	Described in trial design.
Blinding of participants and personnel (performance bias) All outcomes	Low risk	Double‐blind study.
Blinding of outcome assessment (detection bias) All outcomes	Low risk	Clinical‐events committee whose members were unaware of study‐group assignments adjudicated outcomes.
Incomplete outcome data (attrition bias) All outcomes	Low risk	The rates of loss to follow‐up were 0.2% for low‐dose rivaroxaban, 0.3% for normal‐dose rivaroxaban, and 0.3% for placebo.
Selective reporting (reporting bias)	Low risk	All outcomes in the trial registration reported.
Other bias	Low risk	An independent data and safety monitoring committee monitored the trial and reviewed unblinded data.

**Table:** GEMINI‐ACS

***Study characteristics***		
Methods	Randomised, double‐blind, placebo‐controlled trial Study dates: April 2015–October 2016	
Participants	Setting: 371 clinical centres in 21 countries Number randomised/analysed: 3037/3037 Age: median 62 years Sex (male/female): 75%/25%	
Interventions	Experimental group 1 (n = 1519): rivaroxaban 2.5 mg twice daily plus ASA placebo once daily (along with either clopidogrel 75 mg once daily or ticagrelor 90 mg twice daily) for 180–360 days of treatment Experimental group 2 (n = 1518): ASA 100 mg once daily plus rivaroxaban placebo twice daily (along with either clopidogrel 75 mg once daily or ticagrelor 90 mg twice daily) for 180–360 days of treatment Cointerventions: all participants received single antiplatelet therapy with either clopidogrel (43.9%) or ticagrelor (56.1%). Inclusion criteria Age ≥ 18 years Symptoms suggestive of ACS (angina, or symptoms thought to be equivalent) within 48 hours of hospital presentation, or developed ACS while being hospitalised Diagnosis of STEMI or NSTE‐ACS, plus diabetes mellitus or a history of a prior myocardial infarction in participant ≤ 54 years Randomisation within the screening window of 10 days after hospital admission for the index ACS event Acute‐phase treatment for the index ACS, such as intravenous anticoagulant or antiplatelet Maintenance dual antiplatelet therapy with either clopidogrel plus ASA or ticagrelor plus ASA, with the intent to continue the treatment with a platelet adenosine diphosphate P2Y12 receptor antagonist (P2Y12 inhibitor) after randomisation Willingness to provide a pharmacogenomics DNA sample Exclusion criteria Any condition that, in the opinion of the investigator, contraindicates anticoagulant therapy or would have an unacceptable risk Prior stroke of any aetiology or transient ischaemic attack Participant who received thrombolytic therapy as treatment for the index ACS event could not be enrolled in the ticagrelor stratum Anticipated need for chronic administration of omeprazole or esomeprazole concomitantly with clopidogrel Known allergy or intolerance to ASA or rivaroxaban	
Outcomes	Primary outcomes TIMI non‐coronary artery bypass graft (CABG) surgery Clinically significant bleeding (non‐CABG major, minor, or requiring medical attention) up to day 390 Secondary outcomes Global Use of Strategies to Open Occluded Coronary Arteries (GUSTO), Bleeding Academic Research Consortium (BARC), and International Society on Thrombosis and Haemostasis (ISTH) bleeding criteria Composite of cardiovascular death, myocardial infarction, stroke, or definite stent thrombosis All‐cause death Individual component ischaemic endpoints measured throughout the entire study period (ITT population)	
Notes	Funding: Janssen Research & Development and Bayer AG	
***Risk of bias***		
**Bias**	**Authors' judgement**	**Support for judgement**
Random sequence generation (selection bias)	Low risk	Quote: "Participants were randomly assigned (1:1) to either aspirin or rivaroxaban based on a randomisation schedule. Randomisation was balanced by using randomly permuted blocks with size of four and was stratified based on the background P2Y12 inhibitor (clopidogrel or ticagrelor) intended to be used at the time of randomisation".
Allocation concealment (selection bias)	Low risk	Quote: "The interactive web response system assigned a unique treatment code, which dictated treatment assignment for the participant".
Blinding of participants and personnel (performance bias) All outcomes	Low risk	Investigators and participants were masked to treatment assignment. The study drugs (i.e. rivaroxaban and rivaroxaban placebo, aspirin and aspirin placebo) were identical in appearance and were packaged in identical containers.
Blinding of outcome assessment (detection bias) All outcomes	Low risk	All bleeding and ischaemic events were independently adjudicated by a clinical events committee blinded to treatment assignment using previously published criteria.
Incomplete outcome data (attrition bias) All outcomes	Low risk	Participants were analysed according to randomised treatment group, regardless of actual treatment received. A modified ITT analysis was used for bleeding events and included from time of randomisation to 2 days after last dose of study drug. For participants who did not have events, censoring was done at the date of last dose of study drug plus 2 days or the last clinical evaluation date (whichever came first). Only 1 participant was lost to follow‐up during the study period.
Selective reporting (reporting bias)	Low risk	All prespecified outcomes were reported. This study is registered with ClinicalTrials.gov (NCT02293395).
Other bias	Low risk	A data safety monitoring committee reviewed unblinded data throughout the trial. The Duke Clinical Research Institute co‐ordinated the trial, managed the database, and did the secondary and post‐hoc analyses for this report, independent of the sponsors. An international executive committee designed the trial and was responsible for oversight of study conduct and reporting of all results and takes responsibility for the accuracy and completeness of data analyses. The study authors are fully responsible for the study design, data collection, analysis, and interpretation of the results.

**Table:** REDEEM

***Study characteristics***		
Methods	Randomised, double‐blind, placebo‐controlled trial Study dates: March 2008–October 2009	
Participants	Setting: 161 clinical centres in 24 countries Number randomised/analysed: 1878/1878 Age: median 62 years Sex (male/female): 75%/25%	
Interventions	Experimental group 1 (n = 369): dabigatran 50 mg twice daily Experimental group 2 (n = 368): dabigatran 75 mg twice daily Experimental group 3 (n = 406): dabigatran 110 mg twice daily Experimental group 4 (n =347): dabigatran 150 mg twice daily Control group (n = 371): placebo Cointerventions: all participants received aspirin, and 93% received additional clopidogrel. Inclusion criteria ACS with ≥ 1 additional risk factor for cardiovascular complications Exclusion criteria Long‐term treatment with any other oral anticoagulant Severe/disabling stroke within last 6 months Conditions associated with increased bleeding risk Anaemia or thrombocytopenia Severe renal impairment Liver disease Positive pregnancy test	
Outcomes	Primary outcomes Number of participants displaying the composite of major and clinically relevant minor bleeding events (ISTH definition) during total observation time Secondary outcomes Composite of cardiovascular death, non‐fatal myocardial infarction, and non‐haemorrhagic stroke during 6 months of treatment Composite of all‐cause death, non‐fatal myocardial infarction, severe recurrent ischaemia, and non haemorrhagic stroke during 6 months of treatment Individual occurrence of death (cardiovascular and all‐cause), non‐fatal myocardial infarction, severe recurrent ischaemia, and non‐haemorrhagic stroke during 6 months of treatment Number of participants with any reduction of D‐dimer concentration Change from baseline in log10 D‐dimer after 1 and 4 weeks Number of participants with bleeding events during total observation time (ISTH definition of a major bleed and clinically relevant minor bleed). Laboratory analyses: number of participants with possible clinically significant abnormalities (increase or decrease from baseline)	
Notes	Sponsor: Boehringer Ingelheim, Collaborator: Uppsala University	
***Risk of bias***		
**Bias**	**Authors' judgement**	**Support for judgement**
Random sequence generation (selection bias)	Low risk	Randomised via a centralised interactive voice response system (IVRS).
Allocation concealment (selection bias)	Low risk	Randomised via a centralised interactive voice response system (IVRS). The IVRS was re‐programmed to achieve balance between the five groups.
Blinding of participants and personnel (performance bias) All outcomes	Low risk	Double‐blind study.
Blinding of outcome assessment (detection bias) All outcomes	Low risk	All bleeds, deaths, and suspected cases of myocardial infarction, severe recurrent ischaemia, and stroke were evaluated independently by two experienced physicians blinded to study drug assignment.
Incomplete outcome data (attrition bias) All outcomes	Low risk	All bleeds, deaths, and suspected cases of myocardial infarction, severe recurrent ischaemia, and stroke were evaluated independently by two experienced physicians blinded to study drug assignment.
Selective reporting (reporting bias)	Low risk	Outcomes are the same as on the trial registration website (clinicaltrials.gov/ct2/show/NCT00621855).
Other bias	Low risk	Industry‐funded study. Source of funding and conflicts of interests documented. No other sources of bias identified.

ACS: acute coronary syndrome; ASA: acetylsalicylic acid; BAR Bleeding Academic Research Consortium; CABG: coronary artery bypass graft; GUSTO: Global Utilization of Streptokinase and t‐PA for Occluded Coronary Arteries; ISTH: International Society of Thrombosis and Hemostasis; ITT: intention‐to‐treat; LVEF: left ventricular ejection fraction; NSTE‐ACS: non‐ST‐segment elevation acute coronary syndrome; NSTEMI: non‐ST elevation myocardial infarction; STEMI: ST segment elevation myocardial infarction; TIMI: Thrombolysis In Myocardial Infarction.

### Characteristics of excluded studies [ordered by study ID]

**Table:**

Study	Reason for exclusion
Alizadeh 2019	Ineligible study design.
Borst 2018	Ineligible setting.
Devereaux 2018	Ineligible population (surgery).
Duceppe 2018	Ineligible population (surgery).
Euctr 2013a	Ineligible indication.
Euctr 2013b	Ineligible setting.
Gao 2015	Ineligible study design.
Greenberg 2018a	Ineligible indication.
Greenberg 2018b	Ineligible indication.
Kopin 2016	Ineligible indication for anticoagulation (atrial fibrillation).
Lee 2018	Ineligible indication for anticoagulation (atrial fibrillation).
Nct 2012	Ineligible setting.
NCT04333407	Ineligible population.
NCT04688723	Ineligible comparator.
NCT04805710	Ineligible indication for anticoagulation (atrial fibrillation).
NCT04838808	Ineligible population.
Schiele 2018	Ineligible population.
Zannad 2015	Ineligible indication.
Zannad 2018	Ineligible indication.

### Characteristics of ongoing studies [ordered by study ID]

**Table:** NCT03775746

Study name	Can very low dose rivaroxaban in addition to dual antiplatelet therapy (DAPT) Improve thrombotic status in acute coronary syndrome (ACS) ACS (VaLiDate‐R)
Methods	Randomised, parallel assignment, open label
Participants	150 participants
Interventions	Clopidogrel 75 mg tablet Rivaroxaban 2.5 mg tablet Ticagrelor 90 mg tablet
Outcomes	Primary outcomes Change in lysis time (LT) in the 3 treatment groups assessed using the GTT from admission to follow‐up at 30 days, to investigate, in people with recent ACS and who have impaired endogenous fibrinolysis, whether the addition of low dose rivaroxaban to DAPT can improve endogenous thrombotic and fibrinolytic status Secondary outcomes Frequency of further angioplasty (Time frame: 6 months Clinical events including reintervention Frequency of further heart attack, stroke, or death (Time frame: 6 months) Incidence of further major adverse cardiac events
Starting date	8 January 2019
Contact information	East and North Hertfordshire NHS Trust
Notes	Registered at ClinicalTrials.gov (NCT03775746)

ACS: acute coronary syndrome; DAPT: dual antiplatelet therapy; GTT: global thrombosis test.

## Differences between protocol and review

Differences between the published protocol, Al Said 2021, and this current review are as follows.

- We did not investigate heterogeneity through subgroup analyses because we identified few eligible studies.
- We were unable to create net heat plots to visualise inconsistencies between direct and indirect evidence because we identified few eligible studies.
- We were unable to assess the risk of publication bias with funnel plots to explore possible small‐study biases for the primary outcomes because we identified few eligible studies.
- We were unable to analyse recurrent hospitalisation and health‐related quality of life as none of the included studies reported these outcomes.
- We provided in our review the number needed to treat for an additional beneficial outcome (NNTB) and number needed to treat for an additional harmful outcome (NNTH).
